# Functional expression and evaluation of heterologous phosphoketolases in *Saccharomyces cerevisiae*

**DOI:** 10.1186/s13568-016-0290-0

**Published:** 2016-11-15

**Authors:** Alexandra Bergman, Verena Siewers, Jens Nielsen, Yun Chen

**Affiliations:** 1 Department of Biology and Biological Engineering, Chalmers University of Technology, Kemivägen 10, 412 96 Gothenburg, Sweden; 2 Novo Nordisk Foundation Center for Biosustainability, Chalmers University of Technology, Gothenburg, Sweden; 3 Novo Nordisk Foundation Center for Biosustainability, Technical University of Denmark, Lyngby, Denmark

**Keywords:** *Saccharomyces cerevisiae*, Phosphoketolase, Acetyl-CoA, Acetyl-phosphate, Carbon efficiency

## Abstract

Phosphoketolases catalyze an energy- and redox-independent cleavage of certain sugar phosphates. Hereby, the two-carbon (C2) compound acetyl-phosphate is formed, which enzymatically can be converted into acetyl-CoA—a key precursor in central carbon metabolism. *Saccharomyces cerevisiae* does not demonstrate efficient phosphoketolase activity naturally. In this study, we aimed to compare and identify efficient heterologous phosphoketolase enzyme candidates that in yeast have the potential to reduce carbon loss compared to the native acetyl-CoA producing pathway by redirecting carbon flux directly from C5 and C6 sugars towards C2-synthesis. Nine phosphoketolase candidates were expressed in *S. cerevisiae* of which seven produced significant amounts of acetyl-phosphate after provision of sugar phosphate substrates in vitro. The candidates showed differing substrate specificities, and some demonstrated activity levels significantly exceeding those of candidates previously expressed in yeast. The conducted studies also revealed that *S. cerevisiae* contains endogenous enzymes capable of breaking down acetyl-phosphate, likely into acetate, and that removal of the phosphatases Gpp1 and Gpp2 could largely prevent this breakdown. An evaluation of in vivo function of a subset of phosphoketolases was conducted by monitoring acetate levels during growth, confirming that candidates showing high activity in vitro indeed showed increased acetate accumulation, but expression also decreased cellular fitness. The study shows that expression of several bacterial phosphoketolase candidates in *S. cerevisiae* can efficiently divert intracellular carbon flux toward C2-synthesis, thus showing potential to be used in metabolic engineering strategies aimed to increase yields of acetyl-CoA derived compounds.

**Electronic supplementary material:**

The online version of this article (doi:10.1186/s13568-016-0290-0) contains supplementary material, which is available to authorized users.

## Background

The development of technology platforms capable of producing renewable fuels and chemicals is essential for the future of the modern-day society. One such platform are microbial cell factories, which can catalyze the conversion of simple sugars into relevant compounds. The construction of microorganisms for biochemical synthesis is studied within the field of metabolic engineering (Nielsen 2001; Nielsen and Keasling 2016). For example, the industrially well-known and genetically tractable yeast *Saccharomyces cerevisiae* has been successfully engineered to produce high-energy dense fuel candidates such as alkanes, fatty acid ethyl esters (FAEEs) and farnesene (Buijs et al. 2014; de Jong et al. 2014b; Jullesson et al. 2015). These compounds as well as many others within product groups such as food supplements and pharmaceuticals, are derived from the cytosolic two-carbon (C2) precursor molecule acetyl coenzyme A (acetyl-CoA).

**Fig. 1:**
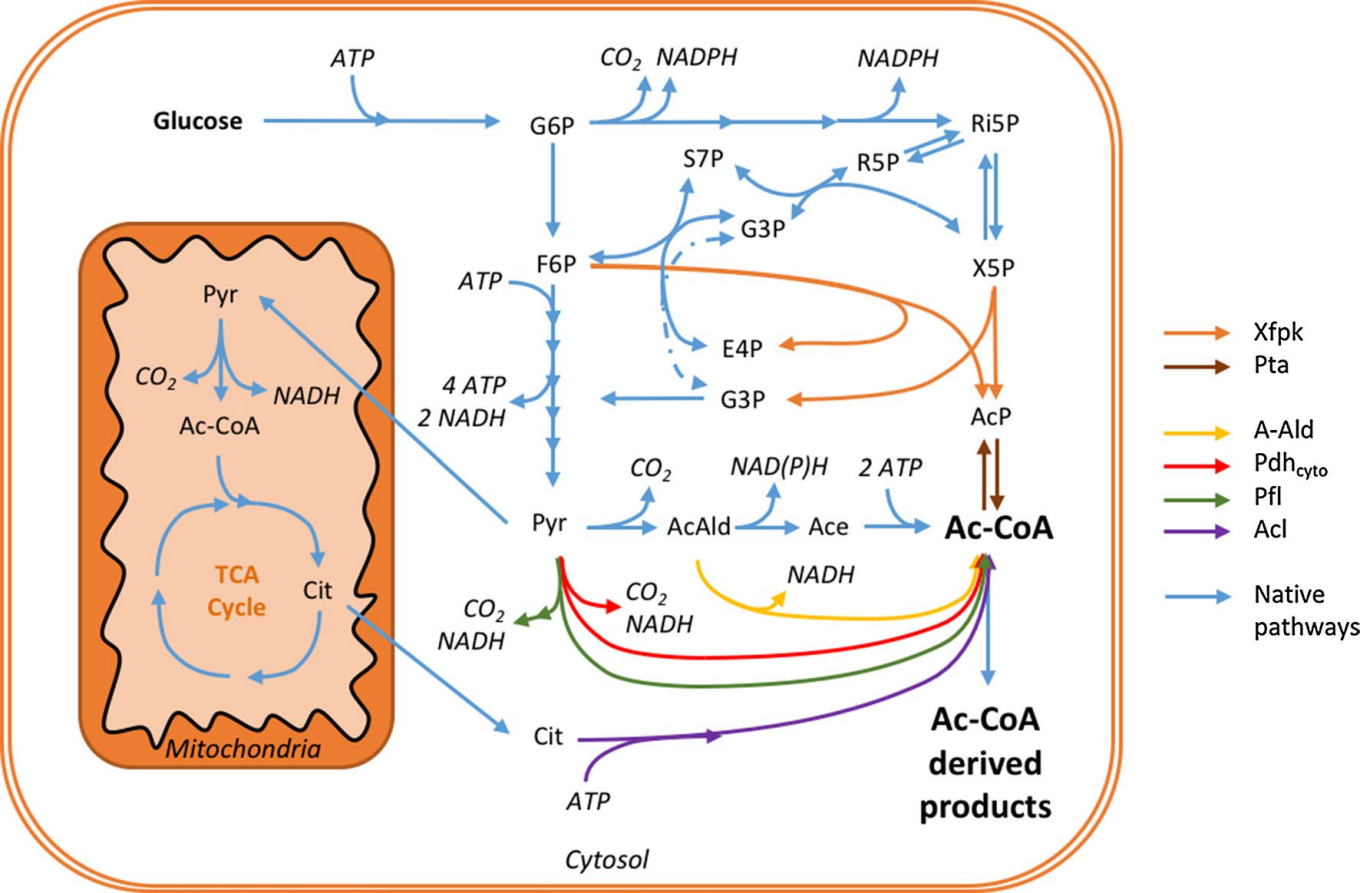
Native and heterologous metabolic pathways for improved cytosolic acetyl-CoA production in *S. cerevisiae*. The native enzymatic pathways are indicated with *light blue arrows*, while the *colored arrows* indicate the alternative pathways that have been evaluated for cytosolic acetyl-CoA production in yeast. *A-Ald* acetylating acetaldehyde dehydrogenase, *Ac-CoA* acetyl-CoA, *AcAld* acetaldehyde, *Ace* acetate, *Acl* ATP citrate lyase, *AcP* acetyl-phosphate, *Cit* citrate, *E4P* erythrose-4-phosphate, *F6P* fructose-6-phosphate, *G3P* glycerol-3-phosphate, *G6P* glucose-6-phosphate, *Pdh*_*cyto*_ cytosolic pyruvate dehydrogenase complex, *Pfl* pyruvate formate lyase, *Pta* phosphotransacetylase, *Pyr* pyruvate, *R5P* ribose-5-phosphate, *Ri5P* ribulose-5-phosphate, *S7P* seduheptulose-7-phosphate, *X5P* xylulose-5-phosphate, *Xfpk* phosphoketolase

The native cytosolic acetyl-CoA forming route in *S. cerevisiae* is thermodynamically efficient, but results in loss of carbon and energy. The biosynthetic reactions involve a decarboxylation step of pyruvate where CO_2_ and acetaldehyde are produced, catalyzed by the cytosolic pyruvate decarboxylase (Pdc) isoenzymes (Krivoruchko et al. 2014). Consequently, through natural yeast metabolism, a maximum of two moles of acetyl-CoA can be produced per mole glucose, while two moles of CO_2_ are lost to the environment. In addition, the natural production route requires two ATP energy equivalents per acetyl-CoA formed, as the activation of acetate by acetyl-CoA synthase (Acs) consumes ATP and releases AMP. Due to these limitations, many strategies have been evaluated to increase cytosolic acetyl-CoA supply in *S. cerevisiae* (van Rossum et al. 2016). In short, the strategies fall into three categories: (1) native pathway upregulation (Shiba et al. 2007), (2) bypass of the ATP-dependent Acs-reaction with heterologous pathways, such as pyruvate formate lyase (Pfl), acetylating acetaldehyde dehydrogenase (A-Ald) and cytosolic pyruvate dehydrogenase (Pdh_cyto_) (Kozak et al. 2014a, b; Zhang et al. 2015) or (3) introduction of the additional acetyl-CoA generating pathways, either an ATP-citrate lyase (Acl) (Feng et al. 2015; Rodriguez et al. 2016; Tang et al. 2013; Zhou et al. 2016) or a two-step pathway based on a phosphoketolase and a phosphotransacetylase (Xfpk/Pta) (de Jong et al. 2014a; Sonderegger et al. 2004). An overview of the native and discussed heterologous pathways for cytosolic acetyl-CoA production in *S. cerevisiae* are shown in Fig. 1.

An upregulation of native biosynthesis does not increase the theoretical yield as it does not affect the energy balance nor the stoichiometry of acetyl-CoA production. An Acs-replacement strategy can positively influence yields as energy may be conserved compared with the native system. However, all of the mentioned heterologous strategies for increased acetyl-CoA formation, except for the phosphoketolase-based pathway, utilize reactions where carbon is or previously was lost as CO_2_. In biorefinery applications both carbon and energy losses lead to a lower theoretical yield of the sought product, which is detrimental when bulk chemical synthesis is considered (Pfleger et al. 2015). An optimal strategy to increase cytosolic acetyl-CoA formation should therefore target both carbon and energy conservation, as in the case of the two-step phosphoketolase-based strategy.

Phosphoketolase (Xfpk; EC 4.1.2.9 and EC 4.1.2.22) plays a key role in the metabolism of heterofermentative and facultative homofermentative lactic acid bacteria (Ganzle 2015) and in the so-called “bifid shunt” of bifidobacteria (Sela et al. 2010). The enzyme acts on the pentose phosphate pathway (PPP) intermediate xylulose-5-phosphate (X5P), and/or on the glycolytic intermediate fructose-6-phosphate (F6P), and produces the two-carbon compound acetyl-phosphate (AcP) while the left-over carbon, glyceraldehyde-3-phosphate or erythrose-4-phosphate, can be recycled in glycolysis and the PPP, respectively (Henard et al. 2015). The AcP forming reactions are thermodynamically favorable [estimated ∆G_R_°′ = −49.9 to −63.2 kJ mol^−1^ (Flamholz et al. 2012)], and both of the substrates can be made available in the cell without any carbon loss (F6P from glycolysis and X5P from non-oxidative PPP reactions). Furthermore, AcP can be converted to acetyl-CoA by another common bacterial enzyme, phosphotransacetylase (Pta; EC 2.3.1.8), without requiring any energy input (Campos-Bermudez et al. 2010). These combined aspects make this metabolic route highly attractive when targeting production of acetyl-CoA derived biochemicals in yeast, and optimal from a carbon-conservation perspective (Bogorad et al. 2013; Henard et al. 2015; van Rossum et al. 2016).

Pta catalyzes a reversible reaction. Although the acetyl-CoA forming reaction is thermodynamically favorable [estimated ∆G_R_°′ = −9.8 kJ mol^−1^ for the forward reaction (Flamholz et al. 2012)], the affinity for the substrate acetyl-CoA is significantly higher than for acetyl-phosphate, e.g. the *Escherichia coli* Pta has about 20 times higher affinity for acetyl-CoA than for acetyl-phosphate (Campos-Bermudez et al. 2010). A system for regulation of the forward and reverse Pta reactions is often present in the native organisms to direct the synthesis and degradation of acetyl-CoA. Therefore, a highly efficient phosphoketolase as well as a drain for the produced acetyl-CoA are required to make the enzyme operate in the direction of acetyl-CoA formation in a heterologous host.

Previous studies have investigated the use of an Xfpk-based pathway for production of acetyl-CoA derived compounds in *S. cerevisiae,* showing that it could significantly improve product titers of FAEE and PHB (de Jong et al. 2014a; Kocharin et al. 2013). In these studies, an Xfpk from the filamentous fungus *Aspergillus nidulans* was used. As phosphoketolase enzymes are widespread in bacterial species (Sanchez et al. 2010), and their specific substrate specificities and kinetics have been shown to differ significantly between species, the efficacy of the recombinant pathway has potential to be increased considerably by investigating new enzyme candidates. Species within the genera *Bifidobacterium*, *Leuconostoc*, *Lactobacillus*, and *Clostridium* have all been shown to harbor functional phosphoketolases (Bogorad et al. 2013; Jeong et al. 2007; Lee et al. 2005; Liu et al. 2012; Meile et al. 2001; Suzuki et al. 2010; Yevenes and Frey 2008), which together with the *Aspergillus* enzyme are examined further in this study.

The aim of this study was to express and evaluate several Xfpk enzyme candidates in the yeast *S. cerevisiae* with the goal to find enzymes that were functionally expressed, had high catalytic activity and varying specificity towards F6P and X5P. In order to achieve this goal, we expressed nine heterologous Xfpks in *S. cerevisiae* and evaluated them in vitro as well as in vivo to determine their activity and investigate how their expression affected yeast physiology. In this way, several bacterial phosphoketolase candidates were identified that displayed higher activity compared with phosphoketolases previously expressed in yeast, and that showed different substrate specificities. These enzymes can be generally utilized for metabolic engineering strategies that attempt to increase production of compounds derived from acetyl-CoA, thus constituting an aid in renewable chemical synthesis.

## Materials and methods

### Phylogenetic analysis

Phosphoketolase gene sequences (see gene nomenclature used in this study and origin of genes in Table 1, and sequence information in Additional file 1: Table S1) were translated and analyzed with the software MEGA7 (Kumar et al. 2016).

**Table 1:** Phosphoketolase nomenclature, organism gene originated from and Gene bank accession number for protein sequences

Gene ID used in this study	Organism	Gene bank accession number
*xfpk*(AN)	*Aspergillus nidulans*	CBF76492.1
*xfpk*(BA)	*Bifidobacterium adolescentis*	KLE27828.1
*xfpk*(BB)	*Bifidobacterium breve*	KND53308.1
*xfpk*(BL)	*Bifidobacterium lactis*	AJD88698.1
*xfpk*(CA)	*Clostridium acetobutylicum*	KHD36088.1
*xfpk*(LM)	*Leuconostoc mesenteroides*	AAV66077.1
*xfpk*(LPP)	*Lactobacillus paraplantarum*	ALO04878.1
*xfpk*(LP1)	*Lactobacillus plantarum*	KRU19755.1
*xfpk*(LP2)	*Lactobacillus plantarum*	KRU18827.1

### Cultivation conditions

For preparation of competent yeast cells, strains were grown in YPD medium (10 g/L yeast extract (Merck Millipore), 20 g/L peptone from meat (Difco), 20 g/L glucose). Selection and cultivation of yeast transformants were conducted on Synthetic Dextrose (SD) medium lacking uracil (SD-URA plates: 6.9 g/L yeast nitrogen base (YNB) without amino acids (Formedium), 0.77 g/L complete supplement mixture without uracil (Formedium), 20 g/L glucose and 20 g/L agar). Counterselection of the *URA3* marker was performed by cultivation on 5-fluoroorotic acid containing plates [5-FOA plates: 6.9 g/L YNB without amino acids (Formedium), 0.77 g/L complete supplement mixture (Formedium), 20 g/L glucose and 0.8 g/L 5-fluoroorotic acid (Sigma)]. All cultivations were performed at 30 °C.

For cultivation of yeast strains carrying plasmids with a *URA3* marker for in vitro and in vivo assays, defined minimal medium was used and prepared according to Verduyn et al. (1992) with modifications (7.5 g/L (NH_4_)_2_SO_4_, 14.4 g/L KH_2_PO_4_, 20 g/L glucose and pH adjusted to 6.5 with 5 M NaOH). Precultures were prepared from single colonies in 3 mL over night at 30 °C, larger cultures were inoculated to an initial OD(600) of 0.1, and cultivated in 20 mL medium (100 mL unbaffled shake flasks) at 30 °C and 200 rpm orbital shaking. Yeast growth rate was monitored by measuring optical density of the cultures at a wavelength of 600 nm (OD(600)).

*Escherichia coli* cultivations were performed at 37 °C in lysogeny broth (10 g/L tryptone (BD Biosciences), 5 g/L yeast extract (Merck Millipore), 10 g/L NaCl or on plates with same composition including the addition of 20 g/L agar). Ampicillin was added when required at a final concentration of 80 µg/mL.

### Plasmid construction

Plasmids were constructed by gap repair in yeast (Oldenburg et al. 1997), using yeast strain CEN.PK 113-5D (*MATa MAL2*-*8* ^*c*^ *SUC2 ura3*-*52*), and named according to Table 2. Yeast transformations were conducted according to the LiAc/SS carrier DNA/PEG method (Gietz and Schiestl 2007). Plasmid amplification was carried out in *E. coli* strain DH5alpha, and preparation of competent *E. coli* cells and transformations were performed according to Inoue et al. (1990). Phusion DNA polymerase (ThermoFisher) was used for amplification of DNA to be used in cloning procedure. Purification of DNA fragments from gel or PCR mixtures were conducted with GeneJet PCR/Gel purification kits (ThermoScientific).

**Table 2:** Plasmids and strains used and constructed in this study

Plasmids	Description	Origin
pSP-GM1	P_*TEF1*_-P_*PGK1*_ bidirectional promoter (2 µm *URA3*)	Chen et al. (2012)
pAB1	pSP-GM1 P_*TEF1*_-*xfpk*(AN)	This study
pAB2	pSP-GM1 P_*TEF1*_-*xfpk*(BA)	This study
pAB3	pSP-GM1 P_*TEF1*_-*xfpk*(BB)	This study
pAB4	pSP-GM1 P_*TEF1*_-*xfpk*(BL)	This study
pAB5	pSP-GM1 P_*TEF1*_-*xfpk*(CA)	This study
pAB6	pSP-GM1 P_*TEF1*_-*xfpk*(LM)	This study
pAB7	pSP-GM1 P_*TEF1*_-*xfpk*(LPP)	This study
pAB8	pSP-GM1 P_*TEF1*_-*xfpk*(LP1)	This study
pAB9	pSP-GM1 P_*TEF1*_-*xfpk*(LP2)	This study

Strains	Genotype	Origin
CEN.PK 113-5D	*MAT*a *MAL2*-*8* ^*c*^ *SUC2 ura3*-*52*	P. Kötter, University of Frankfurt, Germany
5Dgpp1∆	*MAT*a *MAL2*-*8* ^*c*^ *SUC2 ura3*-*52 gpp1*∆	This study
5Dgpp2∆	*MAT*a *MAL2*-*8* ^*c*^ *SUC2 ura3*-*52 gpp2*∆	This study
5Dgpp1∆gpp2∆	*MAT*a *MAL2*-*8* ^*c*^ *SUC2 ura3*-*52 gpp1*∆ *gpp2*∆	This study
AB1	CEN.PK 113-5D pAB1	This study
AB2	CEN.PK 113-5D pAB2	This study
AB3	CEN.PK 113-5D pAB3	This study
AB4	CEN.PK 113-5D pAB4	This study
AB5	CEN.PK 113-5D pAB5	This study
AB6	CEN.PK 113-5D pAB6	This study
AB7	CEN.PK 113-5D pAB7	This study
AB8	CEN.PK 113-5D pAB8	This study
AB9	CEN.PK 113-5D pAB9	This study
AB10	CEN.PK 113-5D pSP-GM1	This study

Synthesized codon optimized (Genescript) phosphoketolase genes (Table 1) were amplified with respective primer pairs (see Additional file 1: Table S2 for all primers used in this study), and the yeast *TEF1* promoter and *ADH1* terminator were amplified from plasmid pSP-GM1 (Chen et al. 2012). All *xfpk* genes were preceded by the Kozak sequence AAAACA to promote translation (Hamilton et al. 1987). A fragment composed of *TEF1* promoter, phosphoketolase gene, and *ADH1* terminator was constructed by fusion PCR according to complementary DNA-sequences added to the primers. pSP-GM1 was linearized with restriction enzyme *Bgl*II (ThermoFisher), purified, and co-transformed together with purified *xfpk* PCR-fusion constructs into CEN.PK 113-5D, and transformants were obtained by growth on SD-URA plates. Colonies were verified with diagnostic PCR. Positive clones were cultivated, plasmids purified with a Yeast plasmid Miniprep I kit (Zymo Research) and *E. coli* was transformed in order to amplify the plasmids. The plasmid sequences were verified with sequencing after plasmid extraction with a GeneJet Miniprep kit (Thermo Scientific). Plasmids with correct insertion and without mutations were selected.

### Strain construction

CEN.PK 113-5D was transformed with the verified plasmids pAB1–pAB9 and the reference plasmid pSP-GM1 and plated on selective SD-URA plates. The strains obtained were purified twice, PCR-verified, and named according to Table 2 to yield strains AB1-9 for phosphoketolase expressing strains and AB10 for the strain harboring pSP-GM1.

Single deletions of *S. cerevisiae* genes *GPP1* and *GPP2* were performed according to PCR-mediated seamless gene deletion principle of Akada et al. (2006). 500 bp recognition sequences up- and downstream of the start-codon of the genes and a 200 bp “direct repeat” sequence located downstream of the lower recognition sequence were amplified from the yeast genome. The *Kluyveromyces lactis* (*Kl*) *URA3* marker was amplified from plasmid pIGS05 (Scalcinati et al. 2012). The four PCR fragments were purified and fused according to complementary DNA-sequences added to primers (see primer sequences used in Additional file 1: Table S2). The fused linear fragments were purified and used in separate transformation procedure of chemically competent yeast strain CEN.PK 113-5D. Transformants were selected on SD-URA plates. Colonies were purified twice and the deletions were verified with PCR. To recover the marker, verified clones were selected on 5-FOA plates and marker loop-out (via direct repeat recombination) was confirmed with PCR. A double deletion was constructed by conducting a *GPP1* deletion in the *GPP2* deletion background. The strains obtained were named according to Table 2, yielding 5Dgpp1∆, 5Dgpp2∆ and 5Dgpp1∆gpp2∆, respectively.

### In vitro phosphoketolase activity assay

Phosphoketolase activity was measured using the ferric hydroxamate method, based on chemical conversion of enzymatically produced acetyl-phosphate into ferric acetyl hydroxamate which can be detected spectrophotometrically, according to a protocol from Meile et al. (2001) with modifications. The substrates D-ribose-5-phosphate (R5P) and d-fructose-6-phosphate (F6P) were used in the assay. R5P was used as a substitute to the natural substrate d-xylulose-5-phosphate (X5P), as R5P can be converted to X5P by the *S. cerevisiae* endogenous enzymes ribose-5-phosphate isomerase and ribulose-5-phosphate 3-epimerase, as indicated by Bogorad et al. (2013). The cofactor thiamin diphosphate was supplied by the crude cell free extract, as exogenous addition was suggested to not have significant effect on phosphoketolase activity (Meile et al. 2001). All chemicals in this section were purchased from Sigma Aldrich if not specified.

Strains AB1-AB10 were cultivated in biological duplicates in 20 mL minimal media to an OD(600) of approximately 1.0. The cells were harvested by centrifugation, washed once with 10 mL of MQ and once with 10 mL of PEB [protein extraction buffer; 50 mM histidine-HCl, 20 mM KH_2_PO_4_–Na_2_HPO_4_, 2 mM dithiothreitol, 1 mM MgSO_4_ (pH 7.0)], before the cell pellet was resuspended in 0.4 mL PEB. All procedures were performed on ice with chilled solutions. Protease inhibitor cocktail was added, after which the cell suspension was transferred to a pre-chilled tube with 0.5 mm glass beads and homogenized using a FastPrep^®^-24 (MP Biomedicals, Santa Ana, CA, USA) (4 cycles of 5.5 m/s for 30 s, 5 min resting on ice in between runs). The homogenized mixture was centrifuged at maximal speed in a table top centrifuge for 10 min at 4 °C, and the crude cell free extract stored on ice. Protein concentrations were determined with a RC DC Protein Assay (Bio-Rad) using bovine serum albumin standards, and protein solutions to be used in assay were diluted to the lowest obtained protein concentration using PEB.

Reactions were carried out in 96-well microtiter plates in a total volume of 75 µL containing KH_2_PO_4_ (33.5 mM, pH 6.5), L-cystein HCl (1.9 mM), NaF (23 mM), ICH_2_COONa (8 mM) and either the substrate R5P (120 mM) or F6P (90 mM) and the crude cell free extract added to start the reaction (22.5 µL). The reactions were incubated at 37 °C for 30 min, after which 75 µL NH_2_OH-HCl (2 M, pH 6.5) was added at room temperature to stop the enzymatic reaction. 10 min later, 50 µL each of Cl_3_CCOOH (0.92 M), HCl (4 M) and FeCl_3_*6H_2_O (0.185 M in 0.1 M HCl) was added to each reaction to generate ferric hydroxamate, visually appearing as brownish coloration. Results were quantified by comparing spectrophotometric measurements at 505 nm with ones obtained from a set of lithium potassium acetyl-phosphate standards using the microplate reader FLUOstar® Omega (BMG Labtech GmbH, Ortenberg, Germany). For each reaction, an identical reaction was conducted in parallel where pure PEB was added instead of dissolved substrate and used to normalize the reads due to protein precipitation in wells. Two biological and two technical replicates were conducted per sample. Phosphoketolase activity was defined in U corresponding to 1 µmol AcP formed per minute per milligram total protein in the reaction.

### In vitro phosphatase activity assay

Strains CEN.PK 113-5D, 5Dgpp1∆, 5Dgpp2∆ and 5Dgpp1∆gpp2∆ were cultivated, cells harvested and cell free extract prepared as indicated in phosphoketolase assay section above. The phosphatase assay was conducted in a similar manner as the ferric hydroxamate method, but in a reaction mixture where exogenous AcP was added as only substrate [protocol modified from a phosphotransacetylase assay by Klotzsch (1969)]. Reactions were carried out in 96-well microtiter plates in a total volume of 75 µL containing Tris–HCl (80 mM, pH 7.4), lithium potassium acetyl-phosphate (20 mM), gluthatione (4 mM), (NH_4_)_2_SO_4_ (12.5 mM) and the crude cell free extract added to start the reaction (22.5 µL). Reactions were incubated for 0–120 min at 37 °C and then processed in the same way as in the phosphoketolase assay.

### HPLC analysis

Fermentation samples were centrifuged, and the supernatant was analyzed using high performance liquid chromatography to quantify the extracellular acetate, glucose, glycerol and ethanol levels. The HPLC system UltiMate® 3000 (Dionex Sunnyvale, CA, USA), equipped with an Aminex® HPX-87H ion exclusion column (Bio-Rad, Hercules, CA, USA), was operated at 45 °C at a flow rate of 0.6 mL/min using 5 mM H_2_SO_4_ as eluent. Glucose and organic acids were detected using a RI and a UV detector, respectively.

## Results

### In vitro assay of phosphoketolase activity reveals functional expression and substrate specificity of examined enzymes

**Fig. 2:**
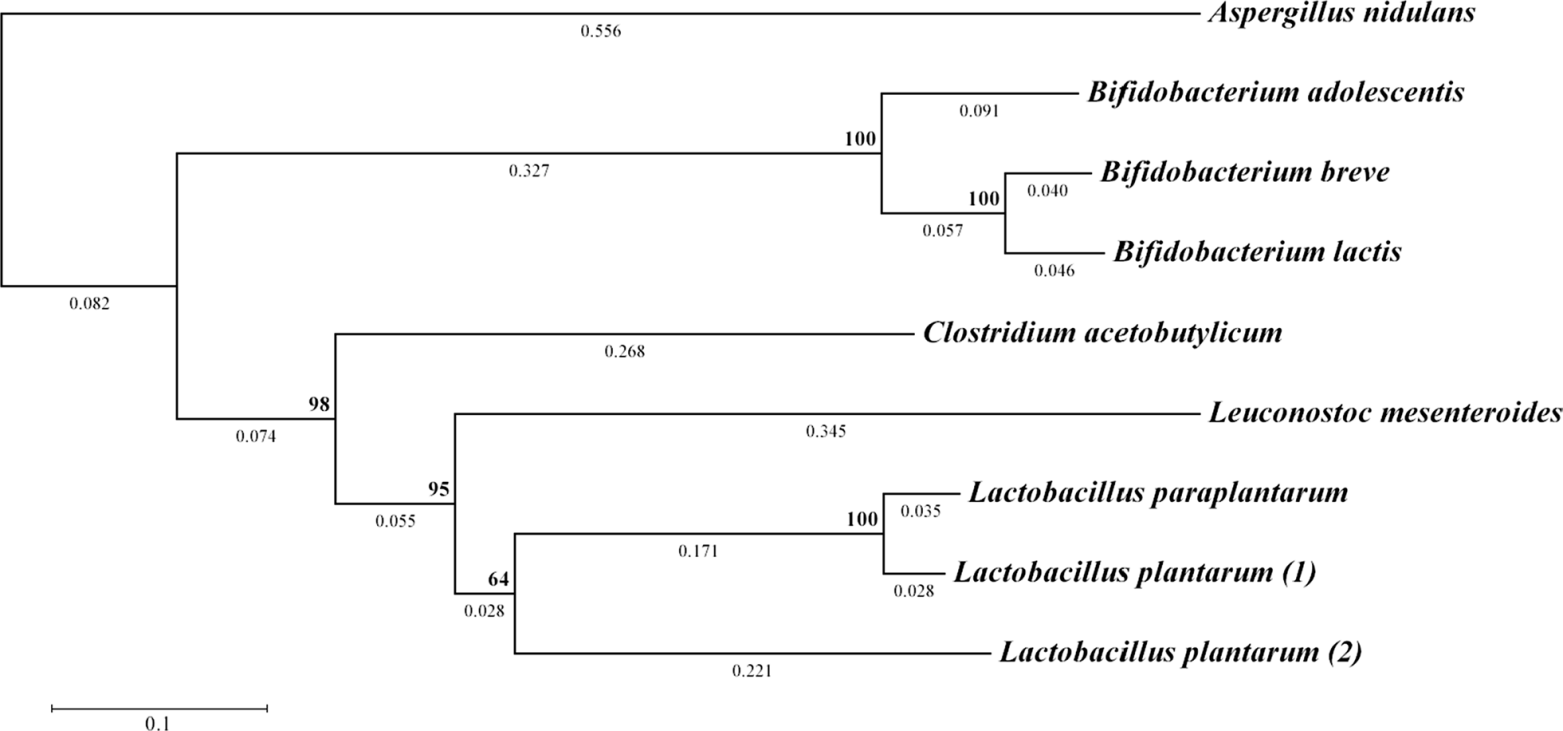
Phylogeny of investigated phosphoketolase candidates. The evolutionary history was inferred using the Neighbor-Joining method. The optimal tree (with the sum of branch length = 2.424) is shown. The percentage of replicate trees in which the associated taxa clustered together in the bootstrap test (1000 replicates) are shown next to the branches. The tree is drawn to scale, with branch lengths (next to the branches) in the same units as those of the evolutionary distances used to infer the phylogenetic tree

Nine phosphoketolase candidates were chosen for this study and their phylogenetic relationships are shown in Fig. 2. In order to select phosphoketolase enzymes that are functionally expressed in yeast and display high enzymatic activity, an in vitro enzyme assay was performed with crude cell extracts from phosphoketolase expressing *S. cerevisiae* strains based on the established ferric hydroxamate method.

**Fig. 3:**
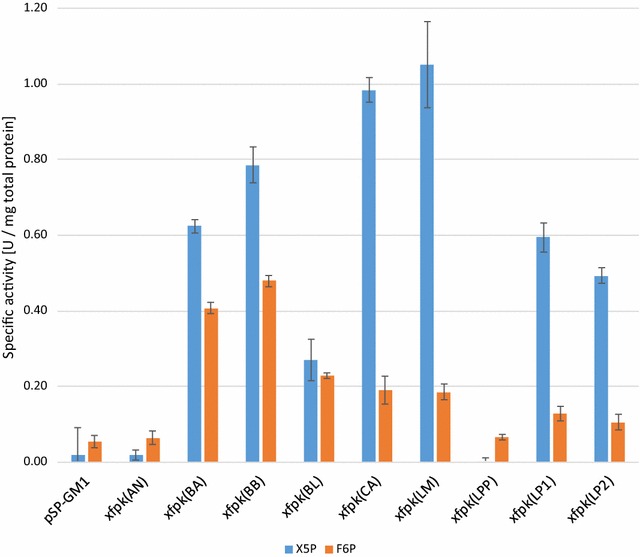
Specific activities of the nine tested Xfpk enzymes with respect to substrates X5P and F6P. The assay was performed using crude cell free extracts. The legends refer to the different *xfpk* genes expressed in strains AB1–AB9. A strain harboring pSP-GM1 (AB10) served as negative control. All *xfpk* candidates except for *xfpk*(AN) and *xfpk*(LPP) displayed significantly higher activity (p < 0.005) when compared to the negative control with respect to both substrates. The results shown are averages from two biological replicates each performed in duplicates; *error bars* indicate the standard deviation

From the spectrophotometric results obtained, the specific activities of the enzyme candidates based on the total protein amount in the crude extracts were calculated, and the results are shown in Fig. 3. Seven out of the nine evaluated phosphoketolase enzymes show significant activity towards both substrates (X5P and F6P) compared to the strain AB10 harboring the empty plasmid pSP-GM1 (p < 0.005), the exceptions being the phosphoketolase candidates from *A. nidulans* and *L. paraplantarum*.

All enzyme candidates with measurable activity show dual substrate specificity, where activity towards X5P appears to be most pronounced for all candidates examined. However, the activity profile for the enzymes originating from the *Bifidobacterium* genus show a higher specificity towards F6P when compared to the results obtained for the enzymes from the Firmicutes phylum (genus *Clostridium*, *Leuconostoc* and *Lactobacillus*). For the *Bifidobacterium* Xfpks, X5P:F6P activity ratios were approximately 3:2 compared to 5:1 in the case of the Firmicutes Xfpks. Also, *S. cerevisiae* appears to form AcP (or another compound that reacts with the enzymatic assay components to yield absorbance at 505 nm) from F6P, as the negative control as well as strains expressing *xfpk*(AN) and *xfpk*(LPP) show a low degree of activity. Thus, the true F6P-phosphoketolases activity might be lower than reported here.

The three candidates with the highest specific activities upon X5P–Xfpk(LM) from *Leuconostoc mesentorides* (1.05 ± 0.11 and 0.19 ± 0.02 U/mg for X5P and F6P, respectively), Xfpk(CA) from *Clostridium acetobutylicum* (0.98 ± 0.03 and 0.19 ± 0.04 U/mg for X5P and F6P, respectively), and Xfpk(BB) from *Bifidobacterium breve* (0.79 ± 0.05 and 0.48 ± 0.02 U/mg for X5P and F6P, respectively)*—*were selected for further studies. The substrate specificities of Xfpk(LM) and Xfpk(CA) appear to be very similar, while the specificity of Xfpk(BB) towards F6P was considerably higher than for the other two candidates, making it an interesting enzyme candidate to evaluate in vivo where intracellular concentrations of F6P should be significantly higher than of X5P during glucose conditions (Wasylenko and Stephanopoulos 2015).

### In vitro assay confirms a strong native enzymatic ability of *S. cerevisiae* to break down acetyl-phosphate

**Fig. 4:**
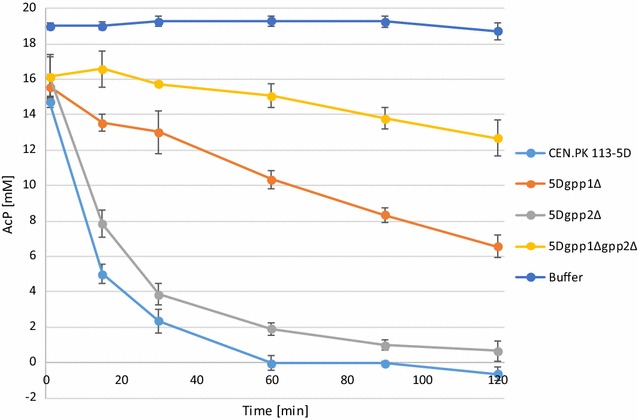
AcP degradation in crude extracts of CEN.PK113-5D and corresponding *GPP1* and/or *GPP2* deletions strains. The legends refer to the strains used to prepare crude cell free extracts. Protein extraction buffer (Buffer) served as a negative control. 20 mM AcP was added in the reaction mixture. Strains carrying deletions show significantly reduced AcP levels when compared to the control strain CEN.PK 113-5D. The results shown are averages from two biological replicates each performed in duplicates; *error bars* indicate the standard deviation

During the setup of the phosphoketolase enzyme assay, it appeared as if *S. cerevisiae* possessed a natural ability to break down acetyl-phosphate, as absorbance of the AcP standard was reduced when it was incubated together with protein extract. Examining literature on the topic made us believe that this breakdown led to a production of acetate (Sonderegger et al. 2004), and was to a large extent catalyzed by two endogenous phosphatases involved in glycerol biosynthesis encoded by genes *GPP1* and *GPP2* (Hawkins et al. 2014). We therefore deleted these genes either alone or in combination and crude cell free extracts prepared thereof were used in an in vitro assay to measure the strains ability to break down acetyl-phosphate over time (Fig. 4).

Gpp1 appears to contribute most to this breakdown, as the AcP samples incubated with cell free extracts derived from the *gpp1*∆ strain retained about 66% of the initial AcP concentration after 60 min, while the corresponding number was 12% for the *gpp2*∆ strain. The double deletion strain reduced the endogenous breakdown activity drastically—93% of initial AcP content was retained after the 60 min incubation (as compared to 0% of the reference strain), and 79% after 120 min. Based on these results and the literature support (Hawkins et al. 2014; Sonderegger et al. 2004), we assumed that in vivo phosphoketolase activity could be assessed by cultivating the wild type (CEN.PK 113-5D) *xfpk*-expressing strains and monitoring acetate levels over time.

### In vivo experiments demonstrate increased acetate formation and reduced growth of phosphoketolase expressing *S. cerevisiae*

In order to confirm the in vitro findings, the three candidate enzymes with the highest specific activities upon R5P–Xfpk(LM), Xfpk(CA) and Xfpk(BB)—and the previously examined phosphoketolase Xfpk(AN) which failed to show activity in the in vitro assay conditions, were selected for a more detailed characterization in vivo. Based on the hypothesis that enzymatically produced AcP is quickly converted endogenously to acetate in wild type yeast, we quantified acetate accumulation in strains carrying the selected *xfpk*-plasmids and the control plasmid pSP-GM1 over time in small scale batch cultures.

**Fig. 5:**
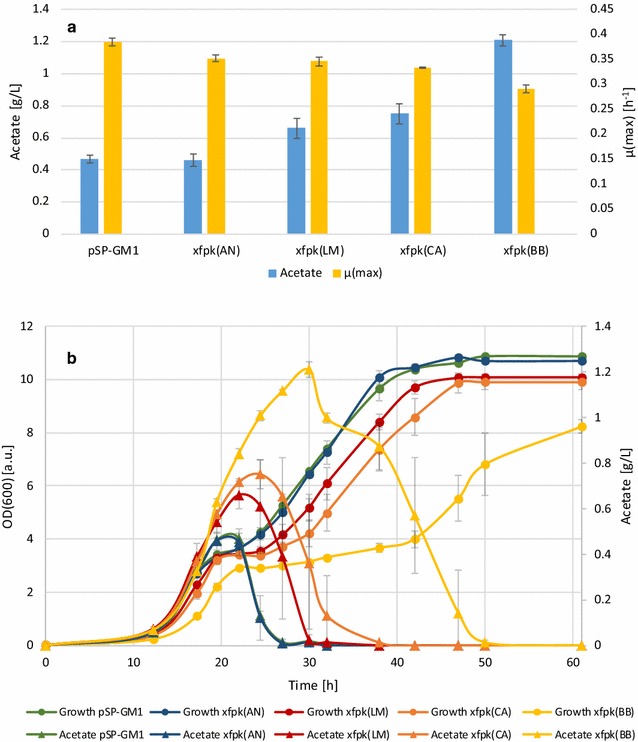
Phosphoketolase activity correlates with increased acetate accumulation and reduced growth rate and biomass formation. **a** Maximal acetate accumulation and maximum specific growth rate measured in cultivations of *xfpk* expressing strains. **b** Acetate accumulation and optical density measurements during whole growth phase. The legends refer to the *xfpk* genes expressed in strains AB1, AB3, AB5 and AB6. pSP-GM1 harboring strain AB10 served as negative control.* Triangles* correspond to acetate measurements and circles optical density. Strains were grown in shake flasks with minimal medium containing 2% glucose. The results shown are averages from three biological replicates; *error bars*s indicate the standard deviation

Figure 5a shows that expression of the three phosphoketolases with high activity as deduced from the in vitro assay significantly (p < 0.05) increased acetate accumulation in vivo compared to the control. The biggest increase was seen for strains expressing *xfpk*(BB), with a maximal level of 1.21 ± 0.04 g/L as compared to the 0.47 ± 0.02 g/L produced by the wild type yeast. The strains expressing *xfpk*(LM) and *xfpk*(CA), corresponding to the phosphoketolases showing similar activity patterns in the in vitro screen, also showed a similar acetate accumulation pattern with maximal titers of 0.66 ± 0.06 and 0.75 ± 0.06 g/L respectively, whereas the strain expressing *xfpk*(AN) did not show any significant difference in acetate accumulation compared to the control. In all strains, the acetate was consumed when cultivations reached stationary phase.

*Xfpk* expression also led to a decreased growth rate (Fig. 5a), decreased biomass formation and an elongated diauxic shift (Fig. 5b). The maximum specific growth rate on glucose (µ_max_) was significantly reduced in all *xfpk* expressing strains (p < 0.01). The biggest decrease was seen for the *xfpk*(BB) expressing strain, where both maximum specific growth rate and final biomass decreased about 25% compared to the control strain.

The time point of the maximum acetate level was reached almost coincidentally with glucose depletion for the control as well as the strain expressing *xfpk*(AN) (Fig. 5b). For the strains expressing *xfpks* generating catalytically active phosphoketolases, however, this time point shifted to 2.5, 5.5 and 8 h after glucose depletion for *xfpk*(LM), *xfpk*(CA) and *xfpk*(BB), respectively.

With regards to other metabolites (Additional file 1: Figure S1), glycerol accumulation during growth on glucose was slightly reduced in the phosphoketolase expressing strains, from 0.82 g/L in the control strain to 0.73 g/L for *xfpk*(BB) and 0.62–0.63 g/L for *xfpk*(LM) and *xfpk*(CA). Ethanol accumulation was not significantly affected by phosphoketolase expression in any of the strains tested, and reached 6.3–6.6 g/L.

## Discussion

This study evaluated yeast expression of phosphoketolases from bacterial origin due to their potential application in metabolic engineering. The phosphoketolase from *A. nidulans* was included as a reference as it had been used beneficially in yeast metabolic strategies previously (de Jong et al. 2014a; Kocharin et al. 2013). From the phylogenetic tree in Fig. 2, it can be seen the selected enzymes in this study fall into two primary groups—enzymes from the *Bifidobacterium* genus and the Firmicutes phylum, both distinct from the fungal phosphoketolase. This is expected, in part because of the evolutionary distance between these groups, but also because phosphoketolase function differs between the two major clusters. The bifidobacterial phosphoketolase has a crucial role in breaking down hexose sugars via the characteristic “bifid shunt” (Pokusaeva et al. 2011), while in lactic acid bacteria, phosphoketolases primarily function to break down pentose sugars (Ganzle 2015). This was also displayed in the in vitro assay as a difference in substrate preference towards F6P and X5P respectively. Indeed, enzymes from the *Bifidobacterium* genus showed higher relative activity towards substrate F6P, compared with the enzymes from the Firmicutes phylum.

The in vitro phosphoketolase results were based on crude cell free extract of strains expressing the heterologous gene candidates. Several of the selected enzymes showed significant activity in the assay, while the two candidates from *A. nidulans* and *L. paraplantarum* did not. As phosphoketolase quantities were not directly measured in this study, it cannot be excluded that low protein production is the cause for this inactivity. Previously reported activities of the candidates upon X5P ranged between 2 and 29 U/mg, whereas in our study this range is between 0.27 and 1.05 U/mg total protein (see comparisons of obtained and reported values in Additional file 1: Table S3). The corresponding ranges for reported F6P specific activity values are 0.1–147.3 U/mg, while we in our study obtained values ranging from 0.11 to 0.48 U/mg. The characterization studies were conducted with purified proteins, with some of the studies reporting purification factors of about 11–24 (Jeong et al. 2007; Meile et al. 2001; Yevenes and Frey 2008), which could explain these large differences. Also, expression differences between yeast and bacterial systems could be a factor explaining the dissimilarities in specific activity measurements.

A general similarity between our obtained data and previously reported values, is that the specific activity towards X5P was higher than towards F6P for all enzymes tested. Previously reported X5P:F6P activity ratios were between 5:1 and 2:1. Also in the present study, the X5P activity was in all instances higher than the F6P activity. In the specific cases, however, differences can be seen, for example for the *Bifidobacterium* species where the obtained ratio between X5P:F6P activity was closer to 3:2, thus showing a higher specificity towards F6P than previously reported. This is in agreement with the fact that the enzyme is solely responsible for F6P degradation in the *Bifidobacterium* species, as discussed above. For all enzymes from the Firmicute phylum the X5P:F6P ratio were approximately 5:1, supporting that their role is to break down pentose sugars in their native hosts.

The candidate that differed most in sequence similarity from the other candidates was Xfpk(AN) from *A. nidulans* (see a percent identity matrix for all protein sequences in Additional file 1: Table S4). The strain expressing *xfpk*(AN) failed to show any significant difference in activity level compared to the negative control in the defined assay conditions. In a previous study, the expression of this gene in combination with a phosphotransacetylase from *Bacillus subtilis* induced a 3.7-fold increase in the production of FAEEs (de Jong et al. 2014a). This could be an indication that the enzymatic activity of Xfpk(AN) is too low to be detected in vitro, possibly due to low sensitivity of the assay. It also shows promise that utilization of the more active phosphoketolase candidates found in this study has potential to increase titers of acetyl-CoA derived products further.

Our study confirmed that *S. cerevisiae* has a strong endogenous capability to break down acetyl-phosphate. We deleted the genes *GPP1* and *GPP2,* encoding two endogenous phosphatases involved in glycerol biosynthesis, as suggested by a patent filed by the company Amyris (Hawkins et al. 2014), which allowed us to determine their individual contribution to the degradation of AcP. Deletions of these two genes may pose a requirement for efficient recombinant pathway flux if a phosphoketolase would be used in combination with a phosphotransacetylase or an acetate kinase, as considerable amounts of carbon otherwise would be processed by Gpp1 and Gpp2, which would reduce the potential energy benefits of the heterologous pathway. However, increased levels of the non-native metabolite AcP might have unexpected impact on yeast physiology. AcP has been suggested to be a key contributor to protein acetylation in *E. coli*, and the compound can acetylate proteins non-enzymatically in vitro (Weinert et al. 2013). As protein acetylation affects gene expression and metabolism in yeast on a global level (Henriksen et al. 2012), it would be beneficial to keep the AcP concentrations in the cell as low as possible.

The native AcP-degrading power of yeast allowed us to estimate phosphoketolase activity in vivo, simply by quantifying acetate production and consumption in small scale shake flask cultivations of the *xfpk* expressing strains. The higher specific activity of Xfpk(BB) towards F6P made it an interesting enzyme to evaluate in vivo, since the intracellular substrate distribution of F6P and X5P differs, and F6P is present at a higher concentration during growth on glucose (Wasylenko and Stephanopoulos 2015). As shown in Fig. 5a, the greatest increase in acetate was indeed seen for the strain expressing *xfpk*(BB), which correlates well with the enzyme’s stronger ability to convert the high-abundant F6P to AcP and erythrose-4-phosphate. Expression of *xfpk*(LM) and *xfpk*(CA) also led to increased acetate accumulation, albeit lower compared to *xfpk*(BB) expression.

*Xfpk* expression furthermore correlated with decreased cellular fitness, such as a reduced growth rate and decreased biomass formation, indicating that expression of these genes leads to some form of cellular stress. The increased acetate formation following *xfpk* expression could explain this behavior, as production of acetic acid will increase ATP consumption when the cell attempts to maintain its intracellular pH levels caused by the proton decoupling effect of the organic acid (Verduyn et al. 1990). A net consumption of ATP will also be the result when acetate produced via the phosphoketolase pathway is metabolized, as its production, contrary to the native pathway, does not generate any ATP. Furthermore, the fact that maximal acetate levels are reached at a later time point in strains expressing the high-activity phosphoketolases compared to the control strain, indicates that the AcP levels in these strains are high after glucose is depleted, which potentially could affect yeast physiology negatively, as mentioned above. It will be important to understand the physiological response in detail in order to counter-act the negative effects of phosphoketolase expression in future studies, for example by studying the effect of an augmented degradation of acetate and AcP.

The results obtained in this study suggest that if a highly efficient phosphoketolase is used in metabolic engineering strategies for improved yields of acetyl-CoA derived products, its expression should be coupled with an efficient channeling of the produced AcP to acetyl-CoA. This can for example be achieved by co-expressing the phosphoketolase with a phophotransacetylase (de Jong et al. 2014a; Sonderegger et al. 2004). Yet, expression of a Pta alone might not be sufficient to relieve problems following phosphoketolase expression in yeast. In a study by Sonderegger et al. (2004), the *xfpk*(BL) was expressed in combination with *pta* from *B. subtilis* in an attempt to increase xylose fermentation and ethanol formation rates. However, *xfpk* expression led to high acetate levels and reduced xylose consumption and ethanol formation rates even in the presence of Pta, which is why their final strategy for increased xylose fermentation was to rely on a weak endogenous phosphoketolase activity in combination with the *B. subtilis* Pta. The deletion of yeast genes *GPP1* and/or *GPP2* in combination with utilization of a more efficient Pta candidate might provide a solution to the problem, as indicated in a patent filed by Amyris, which proposes utilization of Xfpk from *Leuconostoc mesenteroides* in combination with a Pta from *Clostridium kluyveri* for improved production of acetyl-CoA derived chemicals (Hawkins et al. 2014). During the time this manuscript was under review, results were published showing that yeast comprising a deletion of *GPP1*, expression of the above stated heterologous genes together with expression of an acetaldehyde dehydrogenase and a NADH-consuming HMG-CoA reductase produced 25% more of the isoprenoid compound farnesene compared to the reference strain (Meadows et al. 2016), indicating the strong potential of a phosphoketolase based strategy for improved production of acetyl-CoA derived compounds. In general, an efficient system to channel the formed acetyl-CoA to product formation would likely stimulate flux through the heterologous pathway, for example expression of a de-repressed version of acetyl-CoA carboxylase, active under glucose conditions and efficiently forming the fatty acid precursor malonyl-CoA (Shi et al. 2014).

In conclusion, this study has shown that several bacterial heterologous phosphoketolase candidates can be functionally expressed in *S. cerevisiae* and that this can efficiently shift the natural central carbon metabolism, channeling C5 and C6 carbon directly towards C2 synthesis without passing a CO_2_ emitting step. We confirmed that the endogenous metabolism of yeast interferes with the phosphoketolase pathway through a strong, promiscuous ability to degrade AcP, and that deletion of the two native genes *GPP1* and *GPP2* can reduce this conversion. Furthermore, we showed that the expression of phosphoketolases with high catalytic activity led to increased acetate formation and negatively affects growth properties of *S. cerevisiae*. Our analysis provides a valuable resource for identification of suitable phosphoketolase enzymes for metabolic engineering of yeast with the objective to improve acetyl-CoA supply.

## Additional file

**Additional file 1: Figure S1.** Maximal accumulation of ethanol and glycerol in shake flask cultivations of xfpk expressing strains shows no significant change in ethanol levels and low reduction of glycerol levels. **Table S1.** Codon optimized (to *S. cerevisiae*) phosphoketolase gene sequences. **Table S2.** Primers used in this study. **Table S3.** Comparison of obtained and previously reported specific activity values. **Table S4.** Percent identity matrix obtained after multiple sequence alignment of the translated nucleotide sequences used in this study (performed with algorithm CLUSTAL Omega).

## Abbreviations

Acetyl-CoA: acetyl coenzyme A
AcP: acetyl-phosphate
F6P: fructose-6-phosphate
PPP: pentose phosphate pathway
Pta: phosphotransacetylase
Xfpk: phosphoketolase
X5P: xylulose-5-phosphate
