# Supplementary material for: Functional expression and evaluation of heterologous phosphoketolases in Saccharomyces cerevisiae
Source: AMB Express. 2016 Nov 15;6:115. doi: 10.1186/s13568-016-0290-0 (PMC5110461; doi:10.1186/s13568-016-0290-0)
Supplement: Supplementary file 1 — Additional file 1: Figure S1. Maximal accumulation of ethanol and glycerol in shake flask cultivations of xfpk expressing strains shows no significant change in ethanol levels and low reduction of glycerol levels. Table S1. Codon optimized (to S. cerevisiae) phosphoketolase gene sequences. Table S2. Primers used in this study. Table S3. Comparison of obtained and previously reported specific activity values. Table S4. Percent identity matrix obtained after multiple sequence alignment of the translated nucleotide sequences used in this study (performed with algorithm CLUSTAL Omega). [file 13568_2016_290_MOESM1_ESM.docx]

**Supplementary material**

**Figure S1. Maximal accumulation of ethanol and glycerol in shakeflask cultivations of xfpk expressing strains.** The legends refer to the xfpk genes expressed in strains AB1, AB3, AB5 and AB6. pSP-GM1 harboring strain AB10 served as negative control. Strains were grown in shake flasks in minimal medium containing 2% glucose. The results shown are averages from three biological; error bars indicate the standard deviation.

**Table S1. Codon optimized (to S. cerevisiae) phosphoketolase gene sequences.**

| Gene ID and sequence |  |
| --- | --- |
| xfpk(AN)  AAAACAATGCCGGGGGAAGTGATTGAACGTCCTAACCCGGCTCCGAAACCGAGTCATGTGCCTGATCTGGTCGAAAAACTGATTATCCCAGCCCAGAAGACCAAACTGGAAAAAAGTGATTGCGATGCCCTGCATAAATACCGTCGCGCCGCGGCGTATATTGCCGCGGGTCATTGGGGCACCTGTCCGGGCCTGATTCTGGTATATTCTCATCTGAATTATCTGATCAAGAAACAAAACCTGGATATGCTGTACGTGGTCGGTCCGGGTCACGGCGCGCCGGGACTGCTGGCCAGCCTGTGGCTGGAAGGCTCTCTGGGTAAATTCTATCCGCAATATACTAAAGATAAAGAAGGCCTGCATAACCTGATTAGCACGTTTAGCACCAGTGCCGGGCTGCCGAGCCATATTAATGCAGAGACTCCGGGCGCCATTCATGAGGGTGGCGAACTGGGTTATGCTCTGAGCGTCTCGTTTGGTGCGGTGATGGACAACCCGGATCTGATCGTGACCTGCGTGGTTGGTGATGGTGAAGCAGAAACAGGACCTACCGCCACATCGTGGCACGCCATCAAGTATATCGACCCAGCCGAAAGCGGCGCCGTTCTGCCAATCCTGCACGTAAATGGTTTTAAAATTTCCGAACGTACCATTTTCGGGTGCATGGACAATCGCGAGATTGTGTGTCTGTTCACCGGGTATGGTTATCAGGTACGCATTGTCGAAGACCTGGAGGACATTGATAACGATCTGCATTCGGCCATGAGCTGGGCGGTCGAAGAAATTCGTAACATTCAGAAAGCCGCGCGCTCCGGCAAGCCGATTATGAAACCACAGTGGCCGATGATTGTGCTGCGCACGCCGAAAGGTTGGAGTGGACCGAAAGAACTGCATGGTCAGTTTATTGAAGGTAGTTTCCATTCTCATCAGGTCCCTCTGCCGAATGCGAAAAAAGATGATGAAGAGCTGCAGGCCCTGCAAAAATGGCTGTCAAGTTACAAACCGGATGAACTGTTTACGGAAAGCGGCGACGTTATCGACGAAATTCTGAGCATTATTCCATCTGATGATAAAAAGCTGGGCATGCGTCCGGAGGCCTACAAAACTCATCTGCCACCTGACCTGCCTGACTGGCGCCAGTTTTGTGTCAAAAAAGGGGATCAGTTTTCGGCGATGAAAGCGATTGGCAGTTTTATCGATCAGGTTTTTGTGAAAAACCCGCATACCGTGCGCCTGTTTTCTCCAGATGAACTGGAAAGCAATAAGCTGTCAGCCGCGCTGTCCCACACGGGCCGTAACTTTCAGTGGGATGAATTCTCCAACGCCAAAGGGGGTCGCGTGATTGAAGTGCTGTCGGAACACCTGTGTCAGGGGTTCATGCAGGGCTATACCCTGACGGGTCGTACCGGCATTTTTCCTTCCTATGAAAGTTTTCTGGGCATCATCCACACTATGATGGTCCAGTATGCCAAATTTGCGAAAATGGCAAAAGAGACGGCGTGGCACCATGATGTTTCCAGCATTAACTATATTGAAACTTCAACCTGGGCTCGCCAAGAGCATAACGGCTTTAGCCACCAGAACCCGTCGTTTATTGGAGCGGTGCTGAAACTGAAACCGTACGCCGCACGTGTGTATCTGCCGCCTGATGCAAATACGTTTCTGACCACCCTGCATCATTGTCTGAAAAGCAAGAACTATATCAACCTGATGGTTGGGAGCAAACAACCGACACCTGTATACCTGTCACCTGAAGAAGCGGAGTCCCATTGTCGCGCAGGTGCGTCTATCTTCAAATTTTGTAGTACTGACGGCGGTCTGCGTCCGGATGTCGTTCTGGTTGGTATCGGCGTCGAGGTCATGTTCGAAGTTATCAAAGCCGCCGCGATTCTGCGCGAGCGTTGTCCGGAGCTGCGTGTTCGCGTTGTTAACGTTACGGATCTGTTCATTCTGGAAAACGAAGGCGCGCATCCGCACGCTCTGAAACATGAAGCGTTCGATAACCTGTTCACCGAAGATCGCTCTATCCATTTCAATTACCACGGTTACGTGAATGAACTGCAAGGTCTGCTGTTTGGACGCCCACGTCTGGATCGCGCTACGATTAAAGGTTACAAAGAGGAGGGTTCAACTACCACTCCGTTCGACATGATGCTGGTAAACGAAGTTTCACGTTACCATGTAGCGAAAGCGGCTGTAACTGGAGGCGCACGTTTCAACGAAAAAGTCAAACTGCGCCATCAGGAACTGTGCTCAGAGTTTGATCATAACATCGCAGAAACCCGCAAATATATTATGAATAATCATCAGGACCCAGAGGATACCTATAACATGCCGTCGTTTAATTAA | |
| xfpk(BA)  ATGACATCCCCTGTTATCGGTACTCCTTGGAAGAAGTTGAATGCCCCTGTAAGTGAAGAAGCAATAGAAGGTGTAGACAAATACTGGAGAGCTGCAAACTACTTATCTATCGGTCAAATCTATTTGAGATCAAACCCATTGATGAAGGAACCTTTCACAAGAGAAGATGTCAAACATAGATTAGTAGGTCACTGGGGTACTACACCAGGTTTGAACTTTTTAATAGGTCATATCAACAGATTGATAGCTGATCACCAACAAAACACCGTTATTATCATGGGTCCAGGTCATGGTGGTCCTGCCGGTACTGCTCAATCTTACTTAGACGGTACTTACACTGAATACTTCCCAAACATCACCAAGGATGAAGCAGGTTTGCAAAAGTTTTTCAGACAATTTTCTTACCCAGGTGGTATACCTTCACATTATGCTCCAGAAACTCCTGGTTCAATCCACGAAGGTGGTGAATTGGGTTACGCATTATCCCATGCTTATGGTGCCGTTATGAATAACCCAAGTTTATTTGTTCCTGCTATTGTCGGTGACGGTGAAGCCGAAACTGGTCCATTAGCTACAGGTTGGCAATCTAACAAGTTGATCAATCCAAGAACTGATGGTATAGTTTTGCCTATCTTGCATTTGAACGGTTACAAGATCGCTAACCCAACAATCTTGTCCAGAATCAGTGATGAAGAATTACATGAATTTTTCCACGGTATGGGTTATGAACCTTACGAATTTGTCGCTGGTTTCGATAATGAAGACCATTTGTCTATTCACAGAAGATTTGCAGAATTGTTCGAAACTGTTTTCGACGAAATCTGTGATATAAAGGCCGCTGCACAAACCGATGACATGACTAGACCATTCTACCCTATGATCATTTTTAGAACCCCAAAAGGTTGGACTTGCCCTAAGTTCATTGACGGTAAAAAGACAGAAGGTTCTTGGAGATCACATCAAGTTCCATTAGCTTCCGCAAGAGATACCGAAGCTCACTTTGAAGTCTTGAAGAACTGGTTAGAAAGTTACAAGCCTGAAGAATTATTCGATGAAAATGGTGCTGTAAAACCAGAAGTTACAGCTTTTATGCCTACCGGTGAATTGAGAATTGGTGAAAATCCAAACGCTAATGGTGGTAGAATAAGAGAAGAATTGAAATTGCCTAAGTTGGAAGATTACGAAGTAAAGGAAGTTGCAGAATATGGTCATGGTTGGGGTCAATTGGAAGCCACCAGAAGATTAGGTGTTTACACTAGAGACATAATCAAAAATAACCCAGATTCTTTTAGAATTTTCGGTCCTGATGAAACTGCTTCAAACAGATTACAAGCCGCTTACGACGTAACAAATAAGCAATGGGATGCCGGTTATTTGTCTGCTCAAGTTGATGAACACATGGCAGTCACAGGTCAAGTAACCGAACAATTATCAGAACACCAAATGGAAGGTTTCTTGGAAGGTTACTTGTTAACAGGTAGACATGGTATTTGGTCTTCTTATGAATCTTTTGTCCATGTAATCGATAGTATGTTGAATCAACACGCAAAGTGGTTAGAAGCCACTGTTAGAGAAATCCCTTGGAGAAAACCTATTTCCAGTATGAACTTGTTAGTTTCTTCACATGTCTGGAGACAAGACCATAATGGTTTTTCCCACCAAGATCCAGGTGTCACAAGTGTATTGTTGAACAAATGTTTCAACAACGACCACGTTATCGGTATCTATTTCCCTGTCGATTCTAACATGTTGTTGGCCGTTGCTGAAAAGTGCTACAAGTCAACTAACAAGATTAATGCTATCATCGCAGGTAAACAACCAGCAGCCACATGGTTGACCTTAGATGAAGCAAGAGCCGAATTGGAAAAAGGTGCTGCTGAATGGAAGTGGGCCTCCAACGTTAAGAGTAACGACGAAGCTCAAATAGTATTAGCCGCTACTGGTGACGTTCCTACACAAGAAATTATGGCAGCCGCTGATAAGTTGGACGCTATGGGTATTAAGTTTAAAGTTGTCAACGTAGTTGATTTGGTTAAATTACAATCTGCAAAGGAAAATAACGAAGCCTTGTCAGACGAAGAATTTGCAGAATTGTTTACTGAAGATAAACCAGTCTTGTTCGCTTATCATTCCTACGCAAGAGATGTTAGAGGTTTGATCTATGACAGACCAAACCATGATAACTTCAACGTTCACGGTTACGAAGAACAAGGTTCTACCACTACACCTTATGACATGGTTAGAGTCAATAACATCGATAGATACGAATTGCAAGCTGAAGCATTAAGAATGATAGATGCAGACAAATATGCCGACAAGATCAACGAATTGGAAGCCTTTAGACAAGAAGCATTTCAATTCGCAGTTGATAATGGTTATGATCATCCAGACTACACAGATTGGGTATATTCCGGTGTTAACACCAATAAGCAAGGTGCCATTAGTGCTACTGCTGCTACTGCTGGTGACAATGAATAA | |
| xfpk(BB)  ATGACTAACCCTGTAATCGGTACTCCTTGGCAAAAGTTGGATAGACCAGTTTCAGAAGAAGCAATCGAAGGTATGGATAAATATTGGAGAGTTACCAACTATATGTCCATAGGTCAAATCTACTTGAGAAGTAACCCATTGATGAAGGAACCTTTTACTAGAGATGACGTTAAGCATAGATTAGTCGGTCACTGGGGTACTACACCAGGTTTGAACTTCTTGTTGGCCCATATCAACAGATTGATCGCTGATCACCAACAAAACACCGTTTTTATAATGGGTCCAGGTCATGGTGGTCCAGCTGGTACTTCCCAAAGTTATGTTGACGGTACTTACACTGAATACTACCCAAACATAACAAAAGATGAAGCTGGTTTGCAAAAGTTTTTCAGACAATTCTCCTATCCAGGTGGTATCCCTAGTCATTTTGCACCAGAAACCCCTGGTTCAATTCACGAAGGTGGTGAATTGGGTTATGCTTTATCTCATGCTTACGGTGCAGTAATGAATAACCCATCATTGTTTGTTCCTTGTATTATAGGTGACGGTGAAGCCGAAACAGGTCCATTAGCTACCGGTTGGCAATCTAACAAATTGGTCAATCCAAGAACTGATGGTATCGTATTGCCTATCTTGCATTTGAACGGTTACAAGATTGCAAATCCAACAATCTTGGCCAGAATATCTGATGAAGAATTACATGACTTTTTCCGTGGTATGGGTTATCACCCTTACGAATTTGTTGCCGGTTTCGATAATGAAGACCACATGTCTATCCACAGAAGATTCGCTGAATTGTTCGAAACTATCTTCGATGAAATTTGTGACATAAAAGCTGCTGCTCAAACCGATGACATGACTAGACCATTCTACCCTATGTTGATTTTTAGAACTCCAAAGGGTTGGACATGCCCTAAGTTCATCGATGGTAAAAAGACAGAAGGTTCCTGGAGAGCACATCAAGTTCCATTAGCTAGTGCAAGAGATACCGAAGAACACTTTGAAGTCTTGAAAGGTTGGATGGAATCTTACAAGCCTGAAGAATTATTCAATGCAGATGGTTCAATTAAAGATGACGTTACAGCCTTTATGCCAAAGGGTGAATTGAGAATAGGTGCCAATCCTAACGCTAATGGTGGTGTTATCAGAGAAGATTTGAAATTGCCAGAATTAGACCAATATGAAGTAACTGGTGTTAAGGAATACGGTCATGGTTGGGGTCAAGTTGAAGCCCCTAGAGCTTTGGGTGCATATTGTAGAGATATCATTAAAAATAACCCAGACTCCTTTAGAATATTCGGTCCTGATGAAACAGCTAGTAACAGATTGAACGCAACTTATGAAGTAACCGATAAGCAATGGGACAATGGTTACTTGTCTGGTTTAGTTGATGAACACATGGCAGTCACTGGTCAAGTAACAGAACAATTATCAGAACACCAATGCGAAGGTTTCTTGGAAGCATATTTGTTAACAGGTAGACATGGTATTTGGTCTTCATACGAATCTTTTGTACATGTTATCGATTCAATGTTGAACCAACACGCCAAATGGTTAGAAGCTACTGTTAGAGAAATACCTTGGAGAAAGCCTATCTCCAGTGTTAACTTGTTAGTCTCTTCACATGTATGGAGACAAGATCATAATGGTTTTTCTCACCAAGACCCAGGTGTCACATCATTGTTGATTAATAAGACCTTCAATAACGATCACGTTACCAACATCTATTTTGCCACTGACGCTAACATGTTGTTGGCTATCTCTGAAAAGTGCTTCAAGTCAACTAACAAAATCAATGCAATATTCGCCGGTAAACAACCAGCACCTACATGGGTTACCTTGGATGAAGCCAGAGCTGAATTAGAAGCTGGTGCTGCTGAATGGAAATGGGCTTCTAATGCAGAAAATAACGATGAAGTTCAAGTTGTCTTGGCATCCGCCGGTGACGTCCCAACACAAGAATTGATGGCCGCTAGTGATGCTTTGAACAAAATGGGTATTAAGTTTAAAGTAGTTAACGTCGTAGATTTGTTGAAGTTACAATCAAGAGAAAACAACGATGAAGCATTGACTGACGAAGAGTTTACTGAATTGTTTACTGCTGATAAACCAGTATTGTTTGCATATCATTCCTACGCCCAAGATGTTAGAGGTTTGATCTATGATAGACCAAACCATGACAATTTCCACGTTGTCGGTTACAAAGAACAAGGTTCAACCACTACACCTTTTGATATGGTCAGAGTAAATGATATGGACAGATATGCATTGCAAGCAGCCGCTTTGAAGTTAATTGATGCAGACAAATACGCCGATAAGATCGACGAATTAAACGCTTTTAGAAAGAAAGCATTTCAATTCGCAGTCGATAATGGTTATGACATTCCAGAGTTTACTGATTGGGTATACCCTGATGTTAAGGTTGATGAAACACAAATGTTGTCTGCTACTGCTGCTACTGCTGGTGACAATGAATAA | |
| xfpk(BL)  AAAACAATGACAAACCCAGTAATCGGTACACCATGGCAAAAGTTAGATAGACCAGTCTCAGAAGAAGCAATCGAAGGTATGGACAAGTATTGGAGAGTTGCTAACTATATGTCCATAGGTCAAATCTACTTGAGAAGTAACCCATTGATGAAGGAACCTTTTACAAGAGATGACGTTAAGCATAGATTGGTCGGTCACTGGGGTACTACACCAGGTTTGAACTTCTTGTTGGCCCATATCAACAGATTGATCGCTGATCACCAACAAAACACTGTTTTTATTATGGGTCCAGGTCATGGTGGTCCTGCTGGTACAGCACAATCTTATATCGATGGTACCTACACTGAATACTACCCAAACATCACAAAGGATGAAGCTGGTTTGCAAAAGTTTTTCAGACAATTCTCCTATCCAGGTGGTATTCCTAGTCATTTTGCTCCAGAAACCCCTGGTTCTATACACGAAGGTGGTGAATTGGGTTATGCATTATCTCATGCCTACGGTGCTATAATGGATAACCCATCATTGTTCGTTCCTTGTATCATCGGTGACGGTGAAGCTGAAACAGGTCCATTGGCAACCGGTTGGCAATCTAACAAATTAGTCAATCCAAGAACTGATGGTATTGTATTGCCTATATTGCATTTGAACGGTTACAAGATTGCCAATCCAACAATCTTGGCTAGAATATCCGATGAAGAATTGCATGACTTTTTCCGTGGTATGGGTTATCACCCTTACGAATTTGTTGCTGGTTTCGATAATGAAGACCATTTGTCAATACACAGAAGATTCGCAGAATTGTTCGAAACTATCTTCGATGAAATCTGTGACATCAAAGCTGCAGCCCAAACTGATGACATGACAAGACCATTCTACCCTATGTTGATTTTTAGAACTCCAAAGGGTTGGACATGCCCTAAGTTCATCGATGGTAAAAAGACAGAAGGTTCCTGGAGAGCTCATCAAGTTCCATTGGCTAGTGCAAGAGATACCGAAGCACACTTTGAAGTCTTAAAAGGTTGGATGGAATCTTACAAGCCTGAAGAATTGTTCAATGCTGATGGTTCAATTAAAGAAGACGTTACAGCTTTTATGCCAAAGGGTGAATTAAGAATTGGTGCAAATCCTAACGCCAATGGTGGTAGAATCAGAGAAGATTTGAAGTTGCCAGAATTGGACCAATACGAAATCACTGGTGTTAAGGAATACGGTCATGGTTGGGGTCAAGTCGAAGCACCTAGATCATTAGGTGCCTACTGTAGAGATATCATTAAAAATAACCCAGACTCCTTTAGAGTTTTCGGTCCTGATGAAACAGCTAGTAACAGATTGAACGCAACCTACGAAGTTACTAAAAAGCAATGGGATAACGGTTACTTGTCCGCATTAGTAGACGAAAATATGGCCGTTACCGGTCAAGTTGTCGAACAATTGAGTGAACATCAATGCGAAGGTTTCTTAGAAGCTTATTTGTTAACTGGTAGACACGGTATCTGGTCTTCATACGAATCTTTCGTCCATGTAATCGATTCAATGTTGAATCAACACGCTAAATGGTTAGAAGCAACTGTTAGAGAAATACCATGGAGAAAGCCTATCTCCAGTGTCAACTTGTTAGTATCTTCACATGTTTGGAGACAAGATCATAATGGTTTTTCTCACCAAGACCCAGGTGTAACATCAGTTTTGTTGAATAAGACCTTCAATAACGATCATGTTACCAACATCTATTTCGCAACTGACGCCAATATGTTGTTAGCCATTGCTGAAAAGTGCTTCAAGTCAACAAACAAAATCAATGCTATCTTCGCAGGTAAACAACCAGCTGCAACATGGATTACCTTGGATGAAGTTAGAGCTGAATTAGAAGCAGGTGCCGCTGAATGGAAATGGGCCTCTAACGCTAAGTCAAACGATGAAGTACAAGTAGTTTTAGCAGCCGCTGGTGACGTTCCTACTCAAGAAATTATGGCAGCCTCTGATGCTTTGAACAAAATGGGTATTAAGTTTAAAGTCGTAAACGTTGTCGACTTGATCAAATTGCAATCCAGTAAGGAAAACGATGAAGCAATGTCTGATGAAGACTTCGCCGATTTGTTTACAGCTGACAAACCAGTCTTATTTGCATATCATTCATACGCCCAAGATGTTAGAGGTTTGATCTATGATAGACCAAACCACGACAATTTCACTGTAGTTGGTTACAAAGAACAAGGTTCTACCACTACACCTTTTGATATGGTTAGAGTCAACGATATGGACAGATATGCTTTACAAGCAAAAGCCTTGGAATTAATTGATGCCGACAAGTACGCTGATAAGATAAACGAATTGAACGAATTCAGAAAGACCGCATTTCAATTCGCCGTTGATAATGGTTATGACATTCCAGAATTCACTGATTGGGTCTACCCTGACGTAAAAGTTGATGAAACCAGTATGTTATCCGCCACAGCCGCTACAGCAGGTGACAATGAATGA | |
| xfpk(CA):  ATGCAATCCATAATCGGTAAACACAAAGACGAAGGTAAAATAACCCCTGAATACTTGAAAAAGATAGACGCCTACTGGAGAGCAGCCAACTTCATATCAGTTGGTCAATTGTATTTGTTAGATAACCCATTGTTGAGAGAACCATTGAAACCTGAACATTTGAAAAGAAAGGTTGTCGGTCACTGGGGTACTATTCCTGGTCAAAATTTCATCTATGCTCATTTGAACAGAGTCATTAAAAAGTACGATTTGGATATGATATACGTATCTGGTCCAGGTCACGGTGGTCAAGTTATGGTCTCCAATAGTTACTTAGATGGTACATACTCTGAAGTCTACCCAAATGTATCAAGAGACTTAAACGGTTTGAAAAAGTTATGCAAGCAATTTTCTTTCCCTGGTGGTATCTCTTCACACATGGCTCCAGAAACACCTGGTTCAATTAATGAAGGTGGTGAATTGGGTTACTCCTTAGCCCACAGTTTTGGTGCTGTTTTCGATAACCCAGACTTGATTACCGCTTGCGTAGTTGGTGACGGTGAAGCAGAAACCGGTCCTTTAGCAACTTCTTGGCAAGCCAATAAGTTTTTGAACCCAGTAACTGATGGTGCAGTTTTGCCTATCTTGCATTTGAACGGTTACAAGATTTCTAACCCAACAGTTTTGTCTAGGATTCCAAAGGATGAATTAGAAAAGTTTTTCGAGGGTAACGGTTGGAAACCATACTTTGTTGAAGGTGAAGATCCTGAAACTATGCATAAGTTGATGGCCGAAACATTAGACATCGTTACCGAAGAAATCTTGAACATCCAAAAGAATGCTAGAGAAAACAACGATTGTTCAAGACCAAAGTGGCCTATGATTGTTTTAAGAACTCCAAAAGGTTGGACAGGTCCTAAGTTTGTCGATGGTGTACCAAATGAAGGTTCCTTCAGAGCCCATCAAGTTCCTTTGGCTGTCGATAGATATCACACAGAAAACTTGGACCAATTAGAAGAATGGTTGAAGAGTTACAAGCCAGAAGAATTGTTCGATGAAAACTACAGATTGATTCCAGAATTGGAAGAATTAACCCCTAAGGGTAACAAGAGAATGGCTGCAAATTTGCATGCAAACGGTGGTTTGTTATTGAGAGAATTAAGAACTCCAGATTTCAGAGACTATGCCGTAGATGTTCCAACTCCTGGTTCTACAGTTAAACAAGACATGATCGAATTGGGTAAATACGTCAGAGATGTCGTAAAGTTAAACGAAGACACTAGAAACTTCAGAATTTTCGGTCCAGATGAAACAATGTCAAATAGATTGTGGGCTGTTTTTGAAGGTACTAAAAGACAATGGTTGTCCGAAATTAAAGAACCTAACGATGAATTTTTGAGTAACGATGGTAGAATCGTTGACTCTATGTTGTCAGAACATTTGTGCGAAGGTTGGTTGGAAGGTTATTTGTTGACAGGTAGACATGGTTTCTTTGCTTCTTACGAAGCATTTTTAAGAATCGTAGATTCAATGATCACCCAACATGGTAAATGGTTGAAGGTTACTTCTCAATTACCTTGGAGAAAAGACATCGCATCCTTAAATTTGATTGCCACCAGTAACGTTTGGCAACAAGATCATAATGGTTATACTCACCAAGACCCTGGTTTATTGGGTCATATAGTCGATAAAAAGCCAGAAATCGTAAGAGCATACTTGCCAGCTGATGCCAATACTTTATTGGCCGTTTTTGACAAATGTTTGCATACAAAGCACAAGATTAACTTATTGGTCACCTCCAAACATCCAAGACAACAATGGTTGACAATGGATCAAGCTGTTAAGCACGTCGAACAAGGTATTTCTATTTGGGATTGGGCAAGTAATGACAAGGGTCAAGAACCAGATGTTGTCATTGCCTCTTGCGGTGACACACCTACTTTGGAAGCATTGGCCGCTGTTACTATCTTGCATGAACACTTGCCAGAATTAAAAGTAAGATTCGTTAACGTAGTTGATATGATGAAGTTATTGCCAGAAAACGAACATCCTCACGGTTTGTCTGATAAGGACTACAACGCTTTGTTTACTACAGATAAGCCTGTTATATTTGCTTTTCATGGTTTCGCACACTTGATCAATCAATTGACATACCATAGAGAAAACAGAAACTTACATGTTCACGGTTACATGGAAGAAGGTACTATTACCACTCCATTCGATATGAGAGTACAAAATAAGTTGGATAGATTCAACTTAGTTAAGGACGTCGTAGAAAATTTGCCTCAATTGGGTAACAGAGGTGCACATTTGGTTCAATTGATGAACGATAAATTGGTCGAACACAACCAATACATTAGAGAAGTCGGTGAAGACTTACCAGAAATAACAAATTGGCAATGGCATGTATAA | |
| xfpk(LM)  AAAACAATGGCTGATTTCGACTCAAAGGAATACTTAGAATTGGTTGATAAATGGTGGAGAGCTACTAACTACTTGTCCGCTGGTATGATCTTCTTGAAGTCAAACCCATTATTTTCCGTTACTAACACACCTATCAAGGCTGAAGATGTTAAAGTCAAGCCAATTGGTCATTGGGGTACTATATCTGGTCAAACATTCTTGTATGCTCACGCAAACAGATTGATTAACAAATACGGTTTGAATATGTTTTACGTTGGTGGTCCAGGTCATGGTGGTCAAGTAATGGTTACAAACGCTTACTTGGATGGTGCATATACCGAAGACTACCCTGAAATTACTCAAGATATCGAGGGTATGAGTCATTTGTTTAAAAGATTCTCTTTCCCAGGTGGTATTGGTTCACATATGACTGCTCAAACACCTGGTTCATTGCACGAAGGTGGTGAATTGGGTTATTCCTTAAGTCATGCCTTCGGTGCTGTTTTAGATAATCCAGACCAAGTCGCCTTTGCTGTTGTCGGTGACGGTGAAGCAGAAACAGGTCCTTCTATGGCCTCATGGCACTCCATAAAATTTTTGAATGCCAAGAACGATGGTGCTGTTTTACCAGTCTTGGACTTAAATGGTTTCAAAATCTCTAACCCTACAATTTTTAGTAGAATGTCTGATGAAGAAATAACCAAGTTTTTCGAAGGTTTGGGTTACTCACCAAGATTCATTGAAAACGATGACATCCATGATTATGCAACTTACCACCAATTGGCTGCAAACATCTTGGATCAAGCTATCGAAGACATCCAAGCAATCCAAAATGATGCCAGAGAAAACGGTAAATACCAAGACGGTGAAATACCAGCCTGGCCTGTTATTATAGCTAGATTGCCAAAGGGTTGGGGTGGTCCTACACATGATGCATCCAATAACCCAATCGAAAATAGTTTTAGAGCCCATCAAGTTCCATTGCCTTTAGAACAACACGATTTGGCAACTTTACCAGAATTCGAAGACTGGATGAACTCTTACAAGCCTGAAGAATTGTTTAATGCTGATGGTTCATTGAAAGACGAATTAAAGGCAATCGCCCCAAAAGGTGACAAGAGAATGTCCGCCAATCCTATTACAAACGGTGGTGCTGATAGAAGTGACTTGAAGTTACCAAACTGGAGAGAATTCGCAAACGATATAAACGATGACACTAGAGGTAAAGAATTCGCTGATTCAAAGAGAAACATGGACATGGCAACATTGTCTAACTACTTAGGTGCCGTTTCACAATTGAATCCAACCAGATTCAGATTTTTCGGTCCTGATGAAACTATGTCCAATAGATTGTGGGGTTTGTTTAATGTCACCCCTAGACAATGGATGGAAGAAATTAAAGAACCACAAGATCAATTGTTGTCTCCTACTGGTAGAATCATTGACTCACAATTATCCGAACATCAAGCAGAAGGTTGGTTGGAAGGTTATACCTTAACTGGTAGAGTCGGTATTTTCGCTAGTTACGAATCATTTTTGAGAGTAGTTGATACCATGGTAACTCAACATTTCAAGTGGTTGAGACACGCTTCTGAACAAGCATGGAGAAACGATTACCCATCCTTGAACTTAATAGCCACAAGTACCGCTTTCCAACAAGATCATAATGGTTACACACACCAAGACCCAGGCATGTTGACCCATTTGGCAGAAAAGAAATCTAACTTCATCAGAGAATATTTGCCTGCTGATGGTAACTCTTTGTTAGCTGTACAAGAAAGAGCATTTTCAGAAAGACATAAGGTTAATTTGTTGATCGCTTCTAAGCAACCTAGACAACAATGGTTCACTGTAGAAGAAGCAGAAGTTTTGGCCAACGAAGGTTTAAAAATAATCGATTGGGCATCTACTGCCCCATCTTCAGATGTTGACATTACATTTGCTTCAGCAGGTACAGAACCTACCATAGAAACTTTGGCCGCTTTGTGGTTAATCAATCAAGCATTTCCAGATGTCAAGTTTAGATACGTAAACGTCGTAGAATTGTTGAGATTGCAAAAGAAATCTGAACCTAACATGAACGATGAAAGAGAATTAAGTGCTGAAGAATTCAATAAGTACTTCCAAGCAGACACACCAGTTATTTTCGGTTTTCATGCTTACGAAAACTTAATCGAATCATTTTTCTTTGAAAGAAAGTTTACTGGTGACGTCTATGTACACGGTTACAGAGAAGATGGTGACATTACTACAACCTATGATATGAGAGTTTACTCCCATTTGGACAGATTCCACCAAGCCAAAGAAGCAGCCGAAATCTTGAGTGCTAACGGTAAAATAGATCAAGCTGCAGCCGACACTTTCATAGCTAAAATGGATGACACATTGGCAAAGCATTTTCAAGTTACCAGAAATGAAGGTAGAGATATCGAAGAATTCACTGATTGGACCTGGTCTCCATTAAAATAA | |
| xfpk(LPP)  ATGACTACCGATTACTCTTCCCCAGCTTACTTACAAAAGGTCGATAAATACTGGAGAGCCGCTAACTACTTGTCCGTTGGTCAATTATATTTGAAAGACTACCCATTGTTACAACAACCTTTAAAAGCATCTGATGTAAAGGTTCATCCAATATGTCACTGGGGTACTATCGCTGGTCAAAATTCAATCTATGCACATTTGAACAGAGTCATTAACAAATACGGTTTGAAGATGTTCTACGTAGAAGGTCCTGGTCACGGTGGTCAAGTCATGGTATCTAACTCATACTTGGACGGTACATATACCGATATCTATCCAGAAATAACCCAAGATGTTGAGGGTATGCAAAAATTATTCAAGCAATTTTCCTTCCCTGGTGGTGTCGCTAGTCATGCTGCACCAGAAACACCTGGTTCTATTCACGAAGGTGGTGAATTGGGTTATTCCATAAGTCATGGTGTTGGTGCAATCTTAGATAATCCAGACGAAATTGCCGCTGTTGTCGTAGGTGACGGTGAATCTGAAACTGGTCCTTTGGCTACATCCTGGCAAAGTACCAAGTTTATCAATCCAATTAACGATGGTGCAGTTTTGCCTATATTGAATTTGAACGGTTTTAAAATCTCCAACCCAACTATTTTCGGTAGAACAAGTGATGCTAAAATCAAGGAATACTTCGAATCTATGTCATGGGAACCTATTTTCGTCGAAGGTGACGACCCAGAAAAGGTACATCCTGTTTTGGCCAAAGCTATGGATGAAGCAGTTGAAAAGATTAAAGCCATCCAAAAACACGCTAGAGAAAATGATGACGCTACCTTACCAGTTTGGCCTATGATCGTCTTTAGAGCACCAAAAGGTTGGACTGGTCCTAAGTCTTGGGATGGTGACAAAATTGAAGGTTCTTTTAGAGCACATCAAATTCCAATACCTGTTGATCAAAATGACATGGAACACGCAGATGCCTTGGTTGATTGGTTAGAATCTTACCAACCAAAGGAATTGTTTAACGAAGATGGTTCATTAAAGGATGACATCAAGGAAATAATACCAACTGGTGACTCAAGAATGGCAGCCAATCCTATCACCAACGGTGGTGTCGATCCAAAAGCATTGAATTTGCCTAACTTCAGAGATTATGCAGTAGACACTTCCAAGGAAGGTGCCAATGTAAAACAAGATATGTTGGTTTGGAGTGATTACTTAAGAGACGTTATTAAAAAGAATCCAGACAACTTCAGATTGTTCGGTCCTGATGAAACAATGTCTAACAGATTGTACGGTGTTTTTGAAACTACAAACAGACAATGGATGGAAGATATTCATCCAGATTCTGACCAATACGAAGCTGCTGCTGGTAGAGTATTGGATGCCCAATTATCAGAACATCAAGCTGAAGGTTGGTTGGAAGGTTATGTTTTAACTGGTAGACACGGTTTGTTTGCATCCTACGAAGCCTTCTTGAGAGTTGTCGATAGTATGTTGACACAACATTTCAAGTGGTTGAGAAAGGCTAACGAATTAGATTGGAGAAAGAAATACCCATCTTTAAACATCATAGCTGCATCAACTGTTTTCCAACAAGACCATAATGGTTACACCCACCAAGATCCTGGTGCATTGACTCATTTGGCCGAAAAGAAACCAGAATACATAAGAGAATACTTGCCTGCTGACGCAAATACATTGTTAGCTGTCGGTGACGTAATTTTTAGATCACAAGAAAAGATCAACTATGTAGTTACCTCCAAACACCCAAGACAACAATGGTTCAGTATTGAAGAAGCCAAGCAATTGGTTGATAACGGTTTGGGTATCATCGACTGGGCTTCTACTGATCAAGGTTCAGAACCAGATATTGTTTTTGCTGCTGCTGGTACTGAACCTACATTGGAAACCTTAGCCGCTATCCAATTGTTGCATGATTCATTCCCAGAAATGAAGATCAGATTCGTTAACGTCGTAGACATCTTGAAGTTAAGATCACCAGAAAAAGATCCTAGAGGTTTGTCAGATGCAGAATTTGACCATTACTTCACAAAGGATAAGCCAGTTGTCTTTGCCTTCCACGGTTACGAAGATTTGGTTAGAGACATATTTTTCGATAGACATAACCACAACTTATACGTTCATGGTTACAGAGAAAACGGTGACATCACCACTCCATTTGATGTTAGAGTCATGAACCAAATGGATAGATTCGACTTGGCCAAGACTGCTATTGCAGCCCAACCTGCTATGGAAAATACAGGTGCTGCATTTGTTCAATCTATGGATAACATGTTGGCTAAGCATAACGCATACATAAGAGACGCAGGTACAGATTTGCCAGAAGTTAACGATTGGCAATGGAAAGGTTTAAAGTAA | |
| xfpk(LPL1)  AAAACAATGTCTGAAGCCATAAAATCTAAAACCGTTGACTACAGTTCTGACGAATACTTGAAGAGAGTTGACGAATACTGGAGAGCCGCTAATTACATTTCTGTTGGTCAATTGTATTTGTTGAACAACCCATTGTTGAGAGAACCTTTGAAGGCTACCGATGTCAAAGTACATCCAATAGGTCACTGGGGTACTATCGCAGGTCAAAACTTCATCTATGCACATTTGAACAGAGCCATAAACAAATACGGTTTGAACATGTTCTACATCGAAGGTCCTGGTCACGGTGGTCAAGTTATGGTCTCCAATAGTTATTTGGACGGTACATATACCGAAACTTACCCAAAGATCACACAAGATAAGGCTGGTATGAAGAGATTGTTTAAACAATTTTCTTTCCCTGGTGGTGTCGCTTCACATGCAGATCCAAAAACCCCTGGTTCCATTCACGAAGGTGGTGAATTGGGTTACAGTATATTACATGGTGCTGGTGCAGTTTTGGACAATCCAGGTTTAATTGCTGCAACAGTTGTCGGTGACGGTGAATCTGAAACCGGTCCTTTAGCTACTTCATGGCAAGTAAATAAGTTCTTGAACCCAATCACAGATGGTACCGTTTTGCCTATCTTGAATTTGAACGGTTTCAAAATCTCTAACCCAACAGTTTTGTCTAGAGAATCACATGAAGAATTGGAAGATTACTTCAAGGGTTTGGGTTGGGACCCACATTTCGTTGAAGGTACTGATCCTGCCAAAATGCACAAGATAATGGCTGAAGAATTGGATAAAGTCATAGAAGAAATCCATGCTATCAGAAAGAATGCAAAGGATAACAACGACGAATCAAGACCAAAGTGGCCTATGATAGTTTTTAGAGCTCCAAAGGGTTGGACTGGTCCTAAATCTTGGGATGGTGAACCAATCGAAGGTTCATTCAGAGCTCATCAAATCCCAATTCCTGTCGATAGAAATCATATGGAACACGCTGATAAGTTGGTAGACTGGTTGAAGTCTTACAAGCCAGAAGAATTGTTCGATGAAAACGGTACTTTGAAGCCAGAAATTGCCGCTATTATACCTGAAGGTCAAGCTAGAATGGCAGCCAATCCAGTTACAAACGGTGGTAAATTGACAAAGGACTTAATTACCCCTAACATCGATGACTACGCATTGGATAACAAGTCCCATGGTAAAGAAGATGGTAGTGACATGACAGAATTGGGTAAATACATCAGAGATTTGATAGAATTGAATAAGGACAATAAGAACTTTAGAGGTTGGGGTCCAGACGAAACATTGTCAAACAAGTTAGGTGCTGCATTCGAAGATACCAAAAGACAATGGATGGAACCAATTCATGAACCTAATGATGCCTTGTTAGCTCCTCAAGGTAGAATCATTGACTCCATGTTGAGTGAACACATGGATGAAGGCATGTTGGAAGCCTATAACTTAACTGGTAGATACGGTTTCTTTGCTTCTTACGAATCATTTTTGAGAGTAGTTGATTCCATGTTAACCCAACATTTCAAATGGTTGAGAAATAGTCACGAAGAAACTCCATGGAGAGCTGATGTACCTTCTTTGAACGTTATCGCTTCTTCAACCGCATTTCAACAAGACCATAACGGTTACTCCCACCAAGATCCAGGTATAATCAGTCATTTGGCAGAAAAGAAAACTGAATACGTTAGAGCCTACTTACCTGGTGACGCCAATACTTTGATTGCTACATTCGATAAGGCAATCCAATCTAAGCAATTGATCAACTTAATTATAGCATCAAAGCATCCAAGACCTCAATGGTTCACAATGGACGAAGCCAAAAGATTGGTTAGAGATGGTTTAGGTGTCGTAGATTGGGCATCAACTGACCATGGTGAAGAACCAGATGTTGTCTTTGCCACAGCTGGTTCCGAACCTACTACAGAATCCTTGGCCGCTGTTAGTATTTTACACGCTAGATTCCCAGAAATGAAGATCAGATTCATCAACGTAGTTGATTTGTTGAAGTTGAAGAAAGATGACCCAAGAGGTTTGTCTGATGCCGAATTTGACGCTTTCTTTACAAAGGATAAGCCTGTTATCTTCGCATATCATGCCTACGATGACTTAGTCAAGACCATTTTCTTTGATAGACATAACCACAACTTGCATGTTCACGGTTATAGAGAAGAAGGTGACATTACCACTCCATTTGACATGAGAGTCAGAAACGAATTGGATAGATTCCATTTGGTAAAGGCAGCCTTGTTAGCTACTCCTGCATACGCCGAAAAAGGTGCACACGTAATACAAGAAATGAACTCAATCTTGGATAAACATCACGACTATATCAGAGCTGAAGGTACTGATATTCCAGAAGTTGAAAACTGGAAGTGGACAGCATTGAAATAA | |
| xfpk(LP2)  AAAACAATGACTACTGATTACTCATCCCCTGCCTACTTACAAAAGGTCGATAAATACTGGAGAGCCGCTAACTACTTGTCCGTCGGTCAATTATATTTGAAGGACAACCCATTGTTGCAAAGACCTTTAAAAGCTTCTGATGTAAAGGTTCATCCAATAGGTCACTGGGGTACTATCGCTGGTCAAAACTTCATCTATGCACATTTGAACAGAGTCATTAACAAATACGGTTTGAAGATGTTCTACGTAGAAGGTCCAGGTCACGGTGGTCAAGTCATGGTATCTAATTCATACTTGGACGGTACATATACCGATATCTATCCAGAAATAACTCAAGATGTTGAGGGTATGCAAAAGTTGTTTAAACAATTTTCTTTCCCTGGTGGTGTCGCTTCACATGCTGCACCAGAAACACCTGGTTCCATTCACGAAGGTGGTGAATTGGGTTATTCCATAAGTCATGGTGTTGGTGCAATCTTAGATAATCCAGACGAAATTGCCGCTGTTGTCGTAGGTGACGGTGAATCAGAAACTGGTCCTTTGGCTACATCTTGGCAATCAACCAAGTTTATCAATCCAATTAACGATGGTGCAGTTTTGCCTATATTGAATTTGAACGGTTTTAAAATCTCTAACCCAACTATATTCGGTAGAACATCAGATGCTAAGATTAAAGAATACTTCGAATCAATGAACTGGGAACCTATCTTCGTAGAAGGTGACGACCCAGAAAAGGTTCATCCTGCCTTGGCTAAAGCAATGGATGAAGCAGTTGAAAAGATTAAAGCCATCCAAAAACACGCTAGAGAAAATAACGATGCTACTTTACCAGTCTGGCCTATGATAGTTTTTAGAGCACCAAAAGGTTGGACAGGTCCTAAGTCCTGGGATGGTGACAAAATCGAAGGTAGTTTCAGAGCTCATCAAATTCCAATACCTGTTGATCAAAATGACATGGAACACGCCGATGCTTTGGTCGACTGGTTAGAATCCTATCAACCAAAGGAATTGTTTAACGAAGATGGTAGTTTGAAGGATGACATAAAGGAAATAATACCAACTGGTGACTCTAGAATGGCAGCCAATCCTATAACCAACGGTGGTGTCGATCCAAAAGCTTTGAATTTGCCTAACTTCAGAGATTATGCAGTAGACACCTCTAAGGAAGGTGCCAATGTTAAACAAGATATGATCGTCTGGTCAGATTACTTGAGAGACGTTATTAAAAAGAATCCAGACAACTTCAGATTGTTCGGTCCTGATGAAACAATGTCTAACAGATTGTACGGTGTTTTTGAAACTACAAACAGACAATGGATGGAAGATATTCATCCAGATTCCGACCAATACGAAGCACCTGCCGGTAGAGTATTGGATGCCCAATTAAGTGAACATCAAGCTGAAGGTTGGTTGGAAGGTTATGTTTTAACAGGTAGACACGGTTTGTTTGCATCTTACGAAGCCTTCTTGAGAGTTGTCGATTCAATGTTGACCCAACATTTCAAGTGGTTGAGAAAGGCTAACGAATTGGATTGGAGAAAGAAATACCCATCCTTGAACATCATAGCTGCAAGTACTGTTTTCCAACAAGACCATAATGGTTACACCCACCAAGATCCTGGTGCATTGACTCATTTGGCCGAAAAGAAACCAGAATACATAAGAGAATACTTGCCTGCTGACGCAAATACCTTGTTAGCAGTAGGTGACGTTATTTTTAGATCACAAGAAAAGATCAACTACGTAGTTACTTCTAAACACCCAAGACAACAATGGTTCTCAATTGAAGAAGCCAAACAATTGGTCGATAATGGTTTAGGTATAATCGACTGGGCTTCCACTGATCAAGGTAGTGAACCAGATATCGTTTTTGCCGCTGCAGGTACTGAACCTACATTGGAAACCTTAGCCGCTATACAATTGTTGCATGATTCTTTCCCAGAAATGAAGATCAGATTCGTTAACGTCGTAGACATCTTGAAGTTGAGATCCCCAGAAAAAGATCCTAGAGGTTTGAGTGATGCAGAATTCGACCATTACTTCACAAAGGATAAGCCAGTTGTCTTTGCCTTCCACGGTTACGAAGATTTGGTTAGAGACATTTTCTTTGATAGACATAACCACAACTTATACGTTCATGGTTACAGAGAAAACGGTGACATAACCACTCCATTTGATGTTAGAGTCATGAACCAAATGGATAGATTCGACTTGGCCAAGTCTGCTATTGCAGCCCAACCTGCTATGGAAAATACAGGTGCTGCATTTGTTCAATCAATGGATAACATGTTGGCTAAGCATAACGCATACATTAGAGACGCTGGTACAGATTTGCCTGAAGTTAATGACTGGCAATGGAAGGGTTTGAAGTAA | |

**Table S2. Oligonucleotide primers used in this study.**

| No. | Primer ID | Sequence | Amplification Target |
| --- | --- | --- | --- |
| 1 | F-AN | GAAAGAAAGCATAGCAATCTAATCTAAGTTTTAATTACAA-AAAACAATGCCGGGGGAAG | xfpk(AN) |
| 2 | R-AN | CAACCTTGATTGGAGACTTGACCAAACCTCTGGCGAAGAA-TTAATTAAACGACGGCATGTTATAGG | xfpk(AN) |
| 3 | F-BA | GAAAGAAAGCATAGCAATCTAATCTAAGTTTTAATTACAA-AAAACAATGACATCCCCTGTTATCGGTAC | xfpk(BA) |
| 4 | R-BA | CAACCTTGATTGGAGACTTGACCAAACCTCTGGCGAAGAA-TTATTCATTGTCACCAGCAGTAGCA | xfpk(BA) |
| 5 | F-BB | GAAAGAAAGCATAGCAATCTAATCTAAGTTTTAATTACAA-AAAACAATGACTAACCCTGTAATCGGTACTC | xfpk(BB) |
| 6 | R-BB | CAACCTTGATTGGAGACTTGACCAAACCTCTGGCGAAGAA-TTATTCATTGTCACCAGCAGTAGC | xfpk(BB) |
| 7 | F-BL | GAAAGAAAGCATAGCAATCTAATCTAAGTTTTAATTACAA-AAAACAATGACAAACCCAGTAATCGG | xfpk(BL) |
| 8 | R-BL | CAACCTTGATTGGAGACTTGACCAAACCTCTGGCGAAGAA-TCATTCATTGTCACCTGCTG | xfpk(BL) |
| 9 | F-CA | GAAAGAAAGCATAGCAATCTAATCTAAGTTTTAATTACAA-AAAACAATGCAATCCATAATCGGTAAACACA | xfpk(CA) |
| 10 | R-CA | CAACCTTGATTGGAGACTTGACCAAACCTCTGGCGAAGAA-TTATACATGCCATTGCCAATTTGTTATTTC | xfpk(CA) |
| 11 | F-LM | GAAAGAAAGCATAGCAATCTAATCTAAGTTTTAATTACAA-AAAACAATGGCTGATTTCGACTC | xfpk(LM) |
| 12 | R-LM | CAACCTTGATTGGAGACTTGACCAAACCTCTGGCGAAGAA-TTATTTTAATGGAGACCAGGTCCAAT | xfpk(LM) |
| 13 | F-LPP | GAAAGAAAGCATAGCAATCTAATCTAAGTTTTAATTACAA-AAAACAATGACTACCGATTACTCTTCCCCA | xfpk(LPP) |
| 14 | R-LPP | CAACCTTGATTGGAGACTTGACCAAACCTCTGGCGAAGAA-TTACTTTAAACCTTTCCATTGCCAATC | xfpk(LPP) |
| 15 | F-LP1 | GAAAGAAAGCATAGCAATCTAATCTAAGTTTTAATTACAA-AAAACAATGTCTGAAGCCATAAAATC | xfpk(LP1) |
| 16 | R-LP1 | CAACCTTGATTGGAGACTTGACCAAACCTCTGGCGAAGAA-TTATTTCAATGCTGTCCACTTCC | xfpk(LP1) |
| 17 | F-LP2 | GAAAGAAAGCATAGCAATCTAATCTAAGTTTTAATTACAA-AAAACAATGACTACTGATTACTCATCC | xfpk(LP2) |
| 18 | R-LP2 | CAACCTTGATTGGAGACTTGACCAAACCTCTGGCGAAGAA-TTACTTCAAACCCTTCCATTGC | xfpk(LP2) |
| 19 | F-TEF1 | GCACACACCATAGCTTCAAAATG | pSP-GM1 |
| 20 | R-TEF1 | TTGTAATTAAAACTTAGATTAGATTGCTATGC | pSP-GM1 |
| 21 | F-ADH1 | TTCTTCGCCAGAGGTTTGGTCAAGTC | pSP-GM1 |
| 22 | R-ADH1 | GAGAAAGGAAGGGAAGAAAGC | pSP-GM1 |
| 23 | F-GPP1-up | GGAAATCCGTATCATTTTCTCGCATAC | CEN.PK 113-5D |
| 24 | R-GPP1-up | TGCGATGGTTTGTATATTTGCTTTTG | CEN.PK 113-5D |
| 25 | F-GPP1-dw | CTGGGCTGCAGGAATTCGATATCAAGCTTATCGAT-AGTACGGTGAACACTCCATCGAAGTTCCAG | CEN.PK 113-5D |
| 26 | R-GPP1-dw | TTACCATTTCAACAAGTCATCCTTAG | CEN.PK 113-5D |
| 27 | F-DR-GPP1 | TCAGAACAACAAAAGCAAATATACAAACCATCGCA-TTTTCTTTTATTTTTTTGATAAAACTACTAC | CEN.PK 113-5D |
| 28 | R-DR-GPP1 | CATCCAATGCAGACCGATCTTCTACCCAGAATCAC-TCACGGCAGATCATCCGAAGACGACAAAGAAAAG | CEN.PK 113-5D |
| 29 | F-GPP2-up | TCTCAAGTATTTTGGCACCTCGCCCTG | CEN.PK 113-5D |
| 30 | R-GPP2-up | TCCGAATATTGTTTTTATTGTTTTATG | CEN.PK 113-5D |
| 31 | F-GPP2-dw | CTGGGCTGCAGGAATTCGATATCAAGCTTATCGAT-AGTACGGTGAAAAATCCATTGAAGTCCCAGG | CEN.PK 113-5D |
| 32 | R-GPP2-dw | TTACCATTTCAACAGATCGTCCTTAGC | CEN.PK 113-5D |
| 33 | F-DR-GPP2 | TGGAAAAACATAAAACAATAAAAACAATATTCGGA-TCCTCTAAAATCGAACATATTTGAGTAATAATTC | CEN.PK 113-5D |
| 34 | R-DR-GPP2 | CATCCAATGCAGACCGATCTTCTACCCAGAATCAC-ATTTTTCTGAGATGAGACAGAGTCAGAAATAATAG | CEN.PK 113-5D |
| 35 | F-Kl.URA3 | GTGATTCTGGGTAGAAGATCGGTCTG | pIGS05 |
| 36 | R-Kl.URA3 | ATCGATAAGCTTGATATCGAATTCCTGC | pIGS05 |
| 37 | F-Ver.GPP1 | TTGTCTCAGTCCACGGCAATGACATG | CEN.PK 113-5D |
| 38 | R-Ver.GPP1 | ACTGAGGCTGCTAAGAAGTCTGAAAAG | CEN.PK 113-5D |
| 39 | F-Ver.GPP2 | AAATTCCTATGCCTCCTTCGAAAATGG | CEN.PK 113-5D |
| 40 | R-Ver.GPP2 | CATTTTTGGAAGAGAGCAGATTCCC | CEN.PK 113-5D |

**Table S3. Comparison of obtained and previously reported specific activity values of phosphoketolase candidates.**

|  | **Our Study** | | **Previous Study** | | | | | |
| --- | --- | --- | --- | --- | --- | --- | --- | --- |
|  | **Specific Activity [U/mg]** | | **Specific Activity[U/mg]** | | **Purification** | **Host** | **Comment** | **Reference** |
|  | **X5P** | **F6P** | **X5P** | **F6P** |  |  |  |  |
| Xfpk  (AN) | 0.02 ±0.01 | 0.06  ±0.02 | - | - | - | *S. cerevisiae* | Metbolic engineering, no characterization | (de Jong et al. 2014; Kocharin et al. 2013) |
| Xfpk  (BA) | 0.62  ±0.02 | 0.41  ±0.01 | 3.5 | 0.7 | Not reported | *E. coli* | His-tag purification | (Bogorad et al. 2013) |
| Xfpk  (BL) | 0.79  ±0.05 | 0.48  ±0.01 | 29.0 | 14.5 | Not reported | *E. coli* | His-tag purification | (Suzuki et al. 2010) |
| Xfpk  (BB) | 0.27  ±0.05 | 0.23  ±0.01 | - | 4.28 | 11.1x | *B. lactis* | Anion-exchange and size exclusion chromatography purification | (Meile et al. 2001) |
| Xfpk  (LM) | 0.98  ±0.03 | 0.19  ±0.04 | 2.01 | 0.09 | Not reported | *E. coli* | His-tag purification | (Liu et al. 2012) |
| Xfpk  (CA) | 1.05  ±0.11 | 0.19  ±0.02 | - | - | - | *E. coli* | Detection of activity, no characterization | (Lee et al. 2005) |
| Xfpk  (LPP) | -0.02  ±0.03 | 0.07  ±0.01 | - | 147.3 | 23.7x | *E. coli* | GSTrap HP/Factor Xa digestion purification | (Jeong et al. 2007) |
| Xfpk  (LP1) | 0.60  ±0.04 | 0.13  ±0.02 | 4.1 | 1.8 | 11.7x | *E. coli* | Biogel P6/Sephacryl-300 purification | (Yevenes and Frey 2008) |
| Xfpk  (LP2) | 0.49  ±0.02 | 0.11  ±0.02 | - | - | - | *E. coli* | Detection of activity, no characterization | (Yevenes and Frey 2008) |

**Table S4. Percent identity matrix obtained after multiple sequence alignment of the translated phosphoketolase nucleotide sequences.** Analysis performed with algorithm CLUSTAL Omega.

|  | **Xfpk**  **(AN)** | **Xfpk**  **(BA)** | **Xfpk**  **(BL)** | **Xfpk**  **(BB)** | **Xfpk**  **(LM)** | **Xfpk**  **(CA)** | **Xfpk**  **(LP2)** | **Xfpk**  **(LP1)** | **Xfpk**  **(LPP)** |  | **91-100%** |
| --- | --- | --- | --- | --- | --- | --- | --- | --- | --- | --- | --- |
| **Xfpk**  **(AN)** | **100** | 38,74 | 37,4 | 37,94 | 36,91 | 41,26 | 38,61 | 39,68 | 39,68 |  | **71-90%** |
| **Xfpk**  **(BA)** | 38,74 | **100** | 85,82 | 84,85 | 42,89 | 48,15 | 47,39 | 46,34 | 45,96 |  | **51-70%** |
| **Xfpk**  **(BL)** | 37,4 | 85,82 | **100** | 94,91 | 42,77 | 46,88 | 46,75 | 45,57 | 45,19 |  | **31-50%** |
| **Xfpk**  **(BB)** | 37,94 | 84,85 | 94,91 | **100** | 43,4 | 47,01 | 47,52 | 46,34 | 45,96 |  | **0-30%** |
| **Xfpk**  **(LM)** | 36,91 | 42,89 | 42,77 | 43,4 | **100** | 51,72 | 55,54 | 59,47 | 58,96 |  |  |
| **Xfpk**  **(CA)** | 41,26 | 48,15 | 46,88 | 47,01 | 51,72 | **100** | 62,8 | 59,41 | 58,78 |  |  |
| **Xfpk**  **(LP2)** | 38,61 | 47,39 | 46,75 | 47,52 | 55,54 | 62,8 | **100** | 67,94 | 67,3 |  |  |
| **Xfpk**  **(LP1)** | 39,68 | 46,34 | 45,57 | 46,34 | 59,47 | 59,41 | 67,94 | **100** | 98,73 |  |  |
| **Xfpk**  **(LPP)** | 39,68 | 45,96 | 45,19 | 45,96 | 58,96 | 58,78 | 67,3 | 98,73 | **100** |  |  |

**References**

Bogorad IW, Lin TS, Liao JC (2013) Synthetic non-oxidative glycolysis enables complete carbon conservation. Nature 502(7473):693-7. doi:10.1038/Nature12575

de Jong BW, Shi S, Siewers V, Nielsen J (2014) Improved production of fatty acid ethyl esters in Saccharomyces cerevisiae through up-regulation of the ethanol degradation pathway and expression of the heterologous phosphoketolase pathway. Microb Cell Fact 13(1):39. doi:10.1186/1475-2859-13-39

Jeong DW, Lee JM, Lee HJ (2007) Cloning and characterization of a gene encoding phosphoketolase in a Lactobacillus paraplantarum isolated from Kimchi. J Microbiol Biotechnol 17(5):822-9.

Kocharin K, Siewers V, Nielsen J (2013) Improved polyhydroxybutyrate production by Saccharomyces cerevisiae through the use of the phosphoketolase pathway. Biotechnol Bioeng 110(8):2216-24. doi:Doi 10.1002/Bit.24888

Lee JM, Jeong DW, Koo OK, Kim MJ, Lee JH, Chang HC, Kim JH, Lee HJ (2005) Cloning and characterization of the gene encoding phosphoketolase in Leuconostoc mesenteroides isolated from kimchi. Biotechnol Lett 27(12):853-8. doi:10.1007/s10529-005-6718-2

Liu LX, Zhang L, Tang W, Gu Y, Hua Q, Yang S, Jiang WH, Yang C (2012) Phosphoketolase Pathway for Xylose Catabolism in Clostridium acetobutylicum Revealed by C-13 Metabolic Flux Analysis. J Bacteriol 194(19):5413-22. doi:10.1128/Jb.00713-12

Meile L, Rohr LM, Geissman TA, Herensperger M, Teuber M (2001) Characterization of the D-xylulose 5-phosphate/D-Fructose 6-phosphate phosphoketolase gene (xfp) from Bifidobacterium lactis. J Bacteriol 183(9):2929-36. doi:10.1128/Jb.183.9.2929-2936.2001

Suzuki R, Kim BJ, Shibata T, Iwamoto Y, Katayama T, Ashida H, Wakagi T, Shoun H, Fushinobu S, Yamamoto K (2010) Overexpression, crystallization and preliminary X-ray analysis of xylulose-5-phosphate/fructose-6-phosphate phosphoketolase from Bifidobacterium breve. Acta Crystallogr F 66:941-943. doi:10.1107/S1744309110023845

Yevenes A, Frey PA (2008) Cloning, expression, purification, cofactor requirements, and steady state kinetics of phosphoketolase-2 from Lactobacillus plantarum. Bioorg Chem 36(1-3):121-127. doi:10.1016/J.Bioorg.2008.03.002
